# Resequencing and assembly of seven complex loci to improve the *Leishmania major* (Friedlin strain) reference genome

**DOI:** 10.1186/s13071-016-1329-4

**Published:** 2016-02-08

**Authors:** Graciela Alonso, Alberto Rastrojo, Sara López-Pérez, Jose M. Requena, Begoña Aguado

**Affiliations:** Centro de Biología Molecular Severo Ochoa (CSIC-UAM), Universidad Autónoma de Madrid, c/ Nicolás Cabrera, 1, 28049 Madrid, Spain

## Abstract

**Background:**

*Leishmania* parasites cause severe human diseases known as leishmaniasis. These eukaryotic microorganisms possess an atypical chromosomal architecture and the regulation of gene expression occurs almost exclusively at post-transcriptional levels. Accordingly, sequencing of the genome of *Leishmania major*, and subsequently the genome of other related species, was paramount for highlighting these peculiar molecular aspects. Recently, we carried out an analysis of gene expression by massive sequencing of RNA in the *L. major* promastigote, and data derived from that analysis were suggestive of possible errors in the current genome assembly for this *Leishmania* species.

**Results:**

During the analysis by RNA-Seq of the transcriptome for *L. major* Friedlin strain, 163,714 reads could not be aligned with the reference genome. Thus, *de novo* assembly with these reads was carried out and the resulting contigs were further analyzed. After detailed homology searches using available databases, it was postulated that 15 contigs might correspond to genomic sequences lost during the initial genome assembly of the *L. major* Friedlin strain. This was experimentally confirmed by PCR amplification, cloning and sequencing of the new genomic regions. As a result, we have identified seven regions of the *L. major* (Friedlin) genome that were lost during the sequence assembly. This led to the uncovering of six new genes (LmjF.15.1475, LmjF.15.0285, LmjF.24.0765, LmjF.14.0860, LmjF.19.0305, and LmjF.27.2035), and correction of the annotation for two others (LmjF.15.1480 and LmjF.27.2030). Our data suggest that these genomic regions probably collapsed during the genome assembly due to the existence of gene duplications and/or repeated regions surrounding the missed genes.

**Conclusion:**

RNA-seq data helped to reconstruct some genomic regions misassembled during the *L. major* Friedlin genome assembly, which is otherwise quite robust. On the other hand, this study shows that data derived from massive sequencing approaches, including RNA-Seq, should be carefully inspected to improve current genome definition and gene annotations.

**Electronic supplementary material:**

The online version of this article (doi:10.1186/s13071-016-1329-4) contains supplementary material, which is available to authorized users.

## Background

Protists of the genus *Leishmania* are causative agents of a spectrum of human diseases known collectively as leishmaniasis. Disease cases have been reported in 98 countries and three territories on five continents, accounting for the ninth largest disease burden among infectious diseases [1]. Depending on the species of *Leishmania* and host factors, infection of humans may result in different forms of leishmaniasis; the three major forms are cutaneous, mucocutaneous and visceral. *Leishmania major*, causative agent of cutaneous leishmaniasis, was the selected species for determining the first complete genomic sequence of a member of the *Leishmania* genus [2]. In particular, the *L. major* ‘Friedlin’ cloned strain (MHOM/IL/81/Friedlin) was used because a genome physical map was already constructed based on the fingerprint data of 9,216 cosmid clones. The physical link between cosmids and their assignment to specific chromosomes were determined by hybridization analysis in which probes derived from either ends of fingerprint-assembled contigs, expressed sequence tags (ESTs) or known genes were used [3]. Additionally, accuracy of sequence assemblies was assessed by comparison to optical maps for the 36 chromosomes of *L. major* genome [4].

The decoding of the *L. major* genome 10 years ago was a milestone that provided important insights about the gene content and genome architecture of this parasite and paved the way for genome-wide studies [5]. Thus, the completion of the *L. major* genome sequence was the basis for the design of high density oligonucleotide microarrays covering most of the genes; these microarrays have been used to analyze differential gene expression in *L. major* [6] and even in other *Leishmania* species [7]. Also, this genome sequence information has been invaluable for protein identification in *Leishmania* proteomic studies [8], and for analysis of its transcriptome by high-throughput RNA sequencing (RNA-Seq) [9]. In addition, the *L. major* genome sequence has been used as the reference for aligning contigs and creating draft sequences for the genomes of other *Leishmania* species, such as *L. infantum* and *L. braziliensis* [10].

Determination of the precise gene copy number, in loci consisting of multiple tandemly arranged identical genes, is the most challenging issue to resolve using shot-gun sequencing, since sequence reads derived from these repeated genes will collapse into a single contig during genome assembly [11]. Highly expressed proteins such as tubulins, heat shock proteins, proteases, glucose transporters or surface antigens, among others, are encoded by genes present in multiple copies in *Leishmania*. Thus, in this parasite, where gene expression is post-transcriptionally controlled, an increase in the copy number of genes may be envisioned as a direct mechanism to increase the transcript abundance for highly expressed proteins [12]. Nevertheless, it is likely that the real copy number for some of these repeated genes is not accurately registered in current *Leishmania* genome databases. Thus, for example, it has been experimentally demonstrated by Southern blot analysis the existence of six tandemly arranged genes in the *L. major HSP70* locus [13], whereas only two copies are annotated in the *L. major* genome database.

In a recent work, we have used RNA-Seq for establishing a comprehensive transcriptome analysis of the *L. major* promastigote form [9]. For this purpose, RNA-Seq reads were aligned to the *L. major* (Friedlin strain) reference genome and assembled into transcripts. However, a small fraction of the reads could not be aligned to the annotated genome. After filtering these reads by sequence homology with kinetoplast DNA sequences, the remaining reads were *de novo* assembled into contigs. The genomic localization of these contigs in the *L. major* genome was analyzed, and the results indicated that the genomic regions containing these *de novo* assembled sequences were omitted during the original genome assembly due, in most cases, to the presence of long repeated sequences flanking the missed regions.

## Methods

### *Leishmania* culture and DNA isolation

Promastigotes of *L. major* Friedlin strain (MHOM/IL/80/Friedlin; clone V1) were cultured at 26 °C in M199 medium supplemented with 10 % fetal bovine serum, 100 U/ml penicillin G and 0.1 mg/mL streptomycin sulphate. This strain was provided by Dr. Javier Moreno, WHO reference center for leishmaniasis, Instituto de Salud Carlos III (Madrid, Spain). Genomic DNA was obtained from axenic promastigotes using the High Pure PCR Template Preparation Kit (Roche).

### PCR amplification, cloning and sequencing

A list with the oligonucleotides used for amplification and/or sequencing is provided as Additional file 1. For PCR amplifications, the following kits were used: Dream TaqTM Green PCR Master Mix (Fermentas) and Maxime PCR Premix Kit (i-Taq; Intron Biotechnology). The conditions for the PCRs were similar in all cases with slight differences depending on the fragment size and primers melting temperatures (see Additional file 1 for further details). Average amplification conditions were: 95 °C for 5 min followed by 30 cycles of 95 °C for 30 s, 54–62 °C for 30 s and 72 °C for 90 s followed by 72 °C for 10 min. PCR products were gel-purified and cloned into the pCR2.1 vector (Invitrogen). Plasmid DNA was prepared with the AccuPrep Plasmid Mini Extraction Kit (Bioneer).

The “Sanger” sequences were determined using the Big Dye Terminators v3.1 kit (Applied Biosystem, California, USA) by automatic sequencing at the Servicio de Genómica (Parque Científico de Madrid, Universidad Autónoma de Madrid). For sequencing regions with high G + C content the dGTPBigDye Terminator v3.0 Cycle Sequencing Ready Reaction Mix was also used. Sequences were carried out using the ABI Prism 3730 y 3730XL systems.

### Sequence assembly and analysis

RNA-Seq reads of 75-nucleotides were obtained as described elsewhere [9] and are available at the EBI-ENA Sequence Read Archive (SRA) under accession number ERP002077. *De novo* assembling of RNA-Seq reads into contigs were done using the SOAPdenovo software with default parameters [14]. BLAST aligner [15] was used to search sequence homology. Firstly, BLAST alignments against *Leishmania* mitochondrial (kinetoplast) DNA sequences [16] were done. Subsequently, BLAST searches with the sequences of the contigs were performed using the TriTrypDB resources [17]. Thus, contigs where aligned to genome sequences from other *Leishmania* species, including the LV39c5 strain of *L. major*, recently incorporated to the TriTrypDB database. Online BLAST tools from Sanger Institute (https://www.sanger.ac.uk/cgi-bin/blast/submitblast/l_major), TriTrypDB (http://tritrypdb.org/) and NCBI (http://blast.ncbi.nlm.nih.gov/Blast.cgi) databases were also used to search sequences sharing significant identity with the contigs.

After sequence analysis, hypothetical collapsed regions were experimentally demonstrated by PCR. Primers flanking the hypothetical collapse regions or primers mapped to the inner contigs were designed using Primer3Plus [18] and checked for specificity with Primer-BLAST [19] (the primers used are listed in the Additional file 1). Sequences derived from the PCR products were assembled using the online pairwise alignment tool Multalin [20]. Assembled regions were inserted into the current *L.major* Friedlin genome sequence using an in house Python script. RNA-seq reads from Rastrojo et al. [9] were again aligned to the new assembled genome using Bowtie2 [21] with default parameters in order to verify the consistency of the assembled regions. This RNA-seq reads alignment was also used to manually curate the assembled sequences derived from sequencing of PCR products. Additionally, unassembled shotgun reads and unassigned contigs from the *L.major* Friedlin Genome Assembly project from the Sanger Institute (ftp://ftp.sanger.ac.uk/pub/pathogens/Leishmania/major/) were also aligned with Bowtie2 against the new assembled genome and used to confirm the new assembled regions. The sequences generated in this work have been deposited in the ENA, GenBank and DDBJ databases under accession numbers LN874992-LN875024. Accession numbers LN874992-LN874998 contain the sequences of the genomic regions determined by Sanger sequencing; the rest correspond to the *de novo* contigs, assembled from RNA-seq reads.

## Results and discussion

During the analysis by RNA-Seq of the transcriptome for *L. major* Friedlin strain [9], 163,714 reads from a total of 14,656,121 reads could not be aligned with the genome sequence currently available for this strain (TriTrypDB release 6) neither with *Leishmania* mitochondrial DNA sequences [16]. A *de novo* assembling of these reads using the SOAPdenovo software led to the building of 17 contigs higher than 300 nucleotides in length. Our initial hypothesis was that those contigs would represent mitochondrial transcripts, since the currently available sequence for *L. major* mitochondrial genome is not complete [16]. Moreover, many of the *Leishmania* mitochondrial transcripts are extensively edited by uridine addition/deletion [22], and therefore the genome and transcript sequences differ. However, after a detailed analysis, only two out of the 17 contigs were found to correspond to mitochondrial transcripts, based on the existence of significant sequence homology with kDNA maxicircles from other trypanosomatids (GenBank accession numbers: X56015 and FJ203996). Thus, with the remaining 15 contigs, we looked for possible sequence similarities among the different *Leishmania* genome databases at TriTrypDB [17]. Remarkably, ten of them aligned partially with current *L. major* genome (see below), and the remaining aligned with genomic regions from other *Leishmania* species. These findings fueled the idea that these contigs might contain genomic sequences lost during the initial assembling of the *L. major* genome sequence [2]. This hypothesis was experimentally demonstrated by PCR amplification and sequencing. Additionally, unassembled shotgun reads and contigs derived from the *L.major* Friedlin Genome Assembly project (Sanger Institute) were used to confirm our results.

Finally, the analysis allowed the reconstruction of seven genomic regions, which were missed during the *L. major* (Friedlin) genome assembly. Table 1 summarizes the chromosomal location and other features for these regions. The complete sequence of the reconstructed regions is provided in the Additional file 2.

**Table 1:** Reconstructed regions in the *L. major* (Friedlin) genome

Name	Chromosome: location^a^	Size (bp)	New Genes	Modified mRNAs^b^	ENA Accession number	Figure
H150	LmjF.15:598566-601599	8775	LmjF.15.1475	LmjF.15.1480	LN874992	1
H155	LmjF.15:108225-109015	2979	LmjF.15.0285	LmjF.15.0280	LN874993	2
H141	LmjF.24:268817-269759	5109	LmjF.24.0765	LmjF.24.0760	LN874994	3
H147	LmjF.14:341556-342022	3206	LmjF.14.0860	LmjF.14.0850	LN874995	4
H153	LmjF.19:109375-111190	6551	LmjF.19.0305	LmjF.19.0300	LN874996	5
H145	LmjF.36:2301380-2302271	1991	-	LmjF.36.5970, LmjF.36.5980	LN874997	6
H130	LmjF.27:870624-871831	4919	LmjF.27.2035	LmjF.27.2030	LN874998	7

^a^Genomic coordinates according to the current genome assembly (GeneDB database)

^b^GeneDB IDs of the corresponding genes are indicated

### Contigs Cg12150, Cg12126, Cg12149 and Cg12136 mapped to a region containing a gene related to the entry LmjF.15.1480 (region H150)

**Fig. 1:**
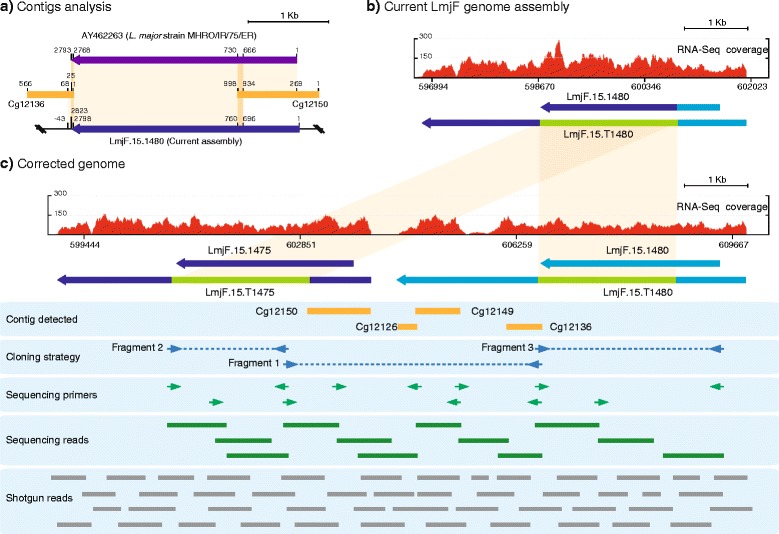
Reconstruction of the *L. major* genomic region containing the locus LmjF.15.1480. **a** Schematic representation of the sequence identity (*orange shadow*) existing between the *de novo* assembled contigs Cg12136 and Cg12150 and the database entries LmjF.15.1480 (TriTrypDB) and gene AY462263 (GenBank). **b** Distribution of RNA-Seq coverage reads and transcript definition (LmjF.15.T1480) on the current annotated genomic region containing the gene LmjF.15.1480. **c** Distribution of RNA-Seq coverage reads and transcript mapping on the corrected genomic region. At the left of panel C is indicated: location of the *de novo* assembled contigs (Contig detected), PCR amplified genomic regions (Cloning strategy), position of the primers used for sequencing purposes (Sequencing primers) of the fragments and the corresponding determined sequences (Sequencing reads), and location of unassigned contigs and unassembled reads obtained in the Sanger *L. major* Genome project (Shotgun reads)

Initial analysis showed that 64 nucleotides at the 3’end of contig Cg12150 (998 nucleotides in length) perfectly aligned within the annotated gene entry LmjF.15.1480 of *L.major* Friedlin (Fig. 1a, b). Also, 68 nucleotides at the 5’ end of contig Cg12136 (566-nucleotides in length) showed a perfect alignment with an *L. major* genomic region covering the 3’ end of the entry LmjF.15.1480 and downstream genomic sequences (Fig. 1a). BLAST search in the GenBank database using contig Cg12150 as query showed that this contig, from position 269 to the end, was 100 % identical to the 5’ end of the entry AY462263 (bases 1–730), a class I phosphodiesterase (PDEB2) transcript described by Johner et al. 2006 [23] in *L. major* (strain MHRO/IR/75/ER) (Fig. 1a). A search in the *L. major* Friedlin TriTrypDB database using the AY462263 sequence indicated that this entry shares high sequence homology with gene LmjF15.1480, which is annotated as a putative cAMP specific phosphodiesterase. Both sequences (AY462263 and LmjF15.1480) are almost identical (99 % of sequence identity) in the region 667–2793 (regarding AY462263), but they are highly divergent in the 5’-end of the genes (Fig. 1a). Hence, we considered the possibility that a gene orthologous to AY462263 would exist in the *L. major* Friedlin genome, but it was missed during the genome assembling process. This hypothesis was reinforced by the finding that contig Cg12150 sequence was covered by unassembled shotgun reads, which remained after assembling of the *L. major* Friedlin genome at Sanger Institute (Shotgun reads, Fig. 1c). In addition, the analysis of the synteny of the equivalent genomic region in other *Leishmania* genomes, available at the TriTrypDB database, showed that the *L. infantum* genome contains two tandemly linked genes homologous to the gene LmjF15.1480 (i.e., LinJ15.1540 and LinJ15.1550). Interestingly, LinJ15.1550 is highly homologous to LmjF15.1480 and, noticeably, LinJ15.1540 is highly homologous to the entry AY462263 [23].

Hence, in order to experimentally test whether a gene homologous to entry AY462263 exists in the *L. major* Friedlin strain, we designed two oligonucleotides to PCR amplify from genomic DNA the region containing the putative missed gene (Fig. 1c, Fragment 1; Cloning strategy). As a result, a genomic PCR product of about 4 kb was obtained. After sequencing, we noticed that not only the contigs Cg12150 and Cg12136 were contained in this region, but two other *de novo* assembled contigs (Cg12126 and Cg12149) also mapped in this region (Fig. 1c). The reconstruction of the genomic fragment indicated that a new gene existed; we named it as LmjF.15.1475 because of its location between genes LmjF.15.1470 and LmjF.15.1480. We postulated that the collapse of this region during the *L. major* genome assembly was due to the high sequence identity existing between genes LmjF.15.1475 and LmjF.15.1480. Oligonucleotides were designed to clone and sequence both genes (Fig. 1c, Fragment 2 and Fragment 3, cloning strategy). The sequences of the three cloned fragments were determined and further checked using the unassembled shotgun reads from the *L. major* genome project available at the Sanger Institute and the *L. major* RNA-Seq data [9] in order to correct sequence errors introduced by Taq polymerase during PCR amplifications. Both genes are almost identical (98 %) in the regions 667–2793 (LmjF.15.1475) and 697–2823 (LmjF.15.1480). Moreover, sequence analysis showed that current annotation for gene LmjF.15.1480 is not accurate. A possible gene duplication and assembly errors in this region were noticed previously by Johner *et al.* [23]. After reconstructing the genomic region, we used the RNA-Seq reads described by Rastrojo *et al.* [9] to define the location of the transcripts corresponding to these genes (Fig. 1b and c; transcripts LmjF.15.T1475 and LmjF.15.T1480). The chimeric nature of the currently annotated LmjF.15.1480 gene was clearly noticed when comparing RNA-Seq reads distribution before (Fig. 1b) and after genomic reconstruction (Fig. 1c).

### The existence of a duplication of gene LmjF.15.0280 was evidenced after analyzing the genomic location of contig Cg12155 (region H155)

**Fig. 2:**
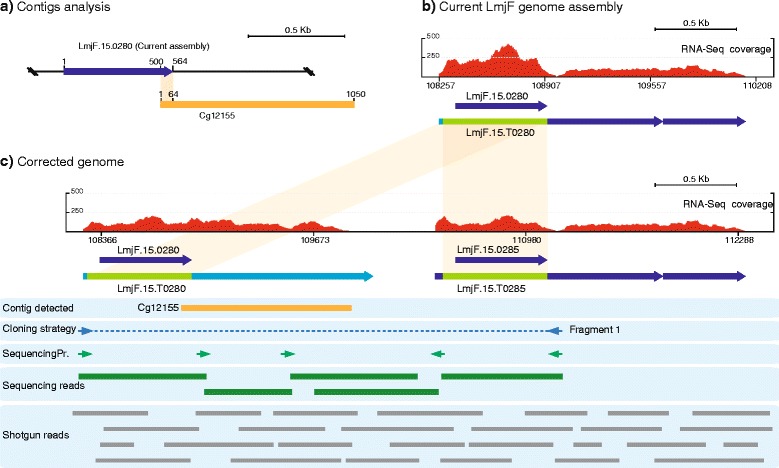
Reconstruction of the *L. major* genomic region containing the locus LmjF.15.0280. **a** Schematic representation of the sequence identity (*orange shadow*) existing between the *de novo* assembled contigs Cg12155 and LmjF.15.1480-containing chromosomal region. **b** Distribution of RNA-Seq coverage reads and transcript (LmjF.15.T0280) definition after alignment with the currently annotated genomic region containing the gene LmjF.15.0280. **c** Distribution of RNA-Seq coverage reads and transcripts mapping on the corrected genomic region. See legend of Fig. 1 for further explanations about figure content

An indication that contig Cg12155 might represent a region lost during *L. major* genome assembly came from the perfect alignment between the first 64 nucleotides of this contig and the 3’-end of gene LmjF.15.0280 (Fig. 2a, b). In the TriTrypDB, this gene is annotated as coding for a putative ribonucleoprotein p18, mitochondrial precursor. Interestingly, in the *L. infantum* and *L. braziliensis* genome databases, two homologous, tandemly repeated genes for LmjF.15.0280 are annotated. Hence, it was hypothesized that *L. major* might also have two tandemly arranged genes. In order to test this hypothesis experimentally, two oligonucleotides were designed on either side of gene LmjF.15.0280 (Fig. 2c, Cloning strategy, Fragment 1). Noticeably, instead of a 789 bp amplicon (according to the current *L. major* Friedlin genome annotation) a ~3 kb amplification product was obtained. Sequencing of the amplified region, confirmed that the complete sequence for contig Cg12155 possessed two nearly identical genes. The gene located at the 3’ end was now renamed as LmjF.15.0285. The coding sequence of the new gene (LmjF.15.0285) was found to be identical to that of gene LmjF.15.0280, although with highly divergent 3’-UTRs. These structural features explain the asymmetric distribution of RNA-Seq reads on current genome assembly (Fig. 2b), where CDS of transcript LmjF.15.T0280 have a 2-fold higher coverage than the 3’- UTR. Hence, after reconstruction of the LmjF.15.0280-LmjF.15.0285 locus (Fig. 2c), the RNA-Seq reads were more homogenously distributed along the CDS and 3’-UTRs in both transcripts.

### Contigs Cg12141 and Cg12160 were mapped at chromosome 24 and served to uncover a new gene (LmjF.24.0765) (region H141)

**Fig. 3:**
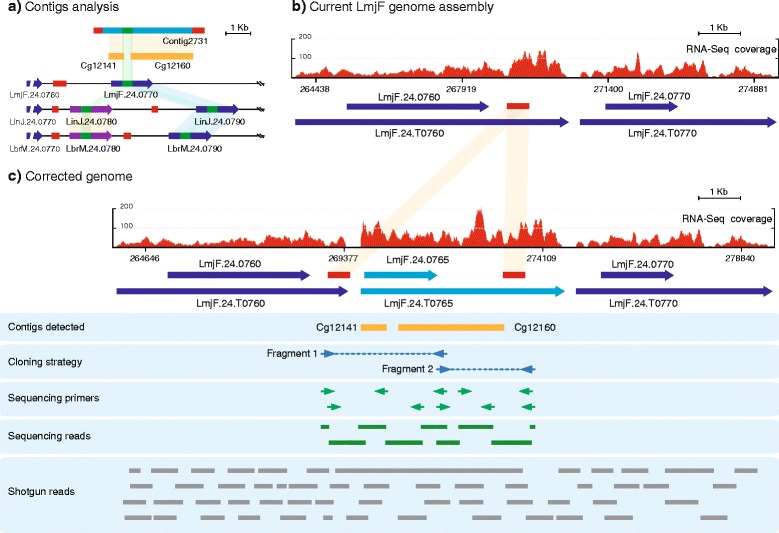
Uncovering of a new gene (LmjF.24.0765) after reconstruction of the intergenic region between genes LmjF.24.0760 and LmjF.24.0770. **a** Schematic representation of the sequence homology existing between the *de novo* contigs (Cg12141 and 12160) and Contig 2731 (*orange shadow*), a contig created during the *L. major* genome sequence project (Sanger Institute database) but not assembled into the final genome annotation. Also, it is shown in light green the blocks of sequence identity existing between the ends of contigs Cg12141 and Cg12160 and the entry LmjF.24.0770. The chromosomal structure of this region in three *Leishmania* species (LmjF: *L. major*; LinJ: *L. infantum*; LbrM: *L. braziliensis*) is also shown. **b** Distribution of RNA-Seq coverage reads and transcripts definition after alignment with the currently annotated genomic region containing the genes LmjF.24.0760 and LmjF.24.0770. **c** Distribution of RNA-Seq coverage reads and transcripts mapping on the corrected genomic region, indicated in light blue, the new gene (LmjF.24.0765) and transcript (LmjF.24.T0765) uncovered before in this chromosomal region. In red are represented the SIDER2 elements. See legend of Fig. 1 for further explanations. The long shotgun read corresponds with the unassembled contig 2731 (Sanger Institute database)

Sixty-three and 62 nucleotides located at the ends of contigs Cg12141 and Cg12160, respectively, were found to align perfectly with an internal region of the gene LmjF.24.0770 (Fig. 3a, b). Additionally, it was found that sequences of these contigs aligned with the *L. infantum* gene LinJ.24.0780, annotated in the TriTrypDB as the gene encoding the malic enzyme. When the sequence of this gene was used for searching among the annotated genes in the *L. major* Friedlin database, a low, but significant, sequence homology with a central portion of gene LmjF.24.0770 was obtained (Fig. 3a). Indeed, the entry LmjF.24.0770 is annotated in TriTrypDB as encoding for malic enzyme; however, based on sequence identity, its putative orthologous in *L. infantum* database would be LinJ.24.0790 rather than LinJ.24.0780. Similarly, the annotated genome of *L. braziliensis* contains two close homologues: LbrM.24.0780 and LbrM.24.0790. On the other hand, a search among the unassigned contigs from the *L. major* genome assembly project at the Sanger Institute resulted in the identification of the Contig 2731, which contains *de novo* assembled contigs Cg12141 and Cg12160, and a complete ORF highly similar (96 % of sequence identity) to that of LinJ.24.0780.

All these data pointed out that a gene orthologous to LinJ.24.0780 might exist in the *L. major* Friedlin genome. Furthermore, as *L. infantum, L. braziliensis,* and *L. major* genomes are largely syntenic [10], it would be expected that this hypothetical gene may be located upstream of gene LmjF.24.0770. However, as the hypothetical and LmjF.24.0770 genes would not have enough sequence conservation (69 % of sequence identity) to explain a collapse during sequence assembly of the *L. major* genome, we analyzed in detail the intergenic region between genes LmjF.24.0760 and LmjF.24.0770 (Fig. 3b), where the hypothetical gene would be located. Interestingly, it was found a Short Interspersed DEgenerated Retroposon (SIDER) element of the family SIDER2. SIDER elements are repeated sequences widespread in the genome of *Leishmania* [24, 25]. Since these elements show remarkable sequence conservation when they are located relatively close in a chromosome [26], it was experimentally addressed that there was a possible assembly collapse due to the existence of two SIDER2 elements flanking the hypothetical gene. Noticeably, repeated elements were detected in both *L. infantum* and *L. braziliensis* flanking genes Linj.42.0780 and LbrM.24.0780 (Fig. 3a).

In order to confirm this hypothesis, oligonucleotides were designed at both sides of the SIDER2 element and within Cg12160 to amplify the whole region into 2 overlapped fragments (Fragment 1 and Fragment 2, Cloning strategy, Fig. 3c). As expected, within the 5,091 bp of the amplified region, the sequences of contigs Cg12141 and Cg12160 were found. Also, the sequence of the hypothesized gene (named LmjF.24.0765) was determined, showing that this gene is the true orthologous to LinJ.24.0780 of *L. infantum*. In addition, the presence of two SIDER2 elements flanking this gene was demonstrated. The sequence of this new region was also confirmed by the alignment of RNA-Seq reads and shotgun reads (Sanger Institute database). Accordingly, a more homogeneous distribution of RNA-Seq reads was observed on transcript LmjF.24.T0760 when they were assembled against the reconstructed region (Fig. 3c) than against current genome assembly (Fig. 3b).

### Contigs Cg12147, Cg12142 and Cg12135 indicated that a duplication of the LmjF.14.0850 ORF exists in *L. major* (Friedlin) (region H147)

**Fig. 4:**
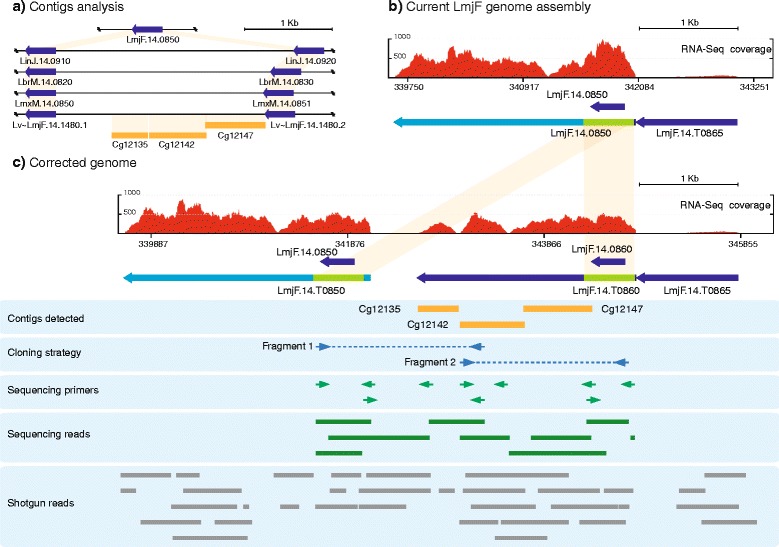
The gene LmjF.14.0850 is tandemly duplicated in the *L. major* Friedlin genome. **a** Comparison of the genomic regions containing gene LmjF.14.0850 (and its orthologous) in five *Leishmania* species (LmjF: *L. major* strain Friedlin; LinJ: *L. infantum*; LbrM: *L. braziliensis*; LmxM: *L. mexicana;* Lv: *L. major* strain LV39). The locations of the *de novo* assembled contigs Cg12135, Cg12142 and Cg12147 regarding the *L. major* LV39 genomic region (contig KB217907; TriTrypDB database) are shown. In orange shadow are indicated areas of high sequence identity. **b** Distribution of RNA-Seq coverage reads and transcript definition after alignment with the currently annotated genomic region containing the gene LmjF.14.0850. **c** Distribution of RNA-Seq coverage reads and transcript mapping on the corrected genomic region. The duplication of the gene LmjF.14.0850 (LmjF.14.0860) is indicated. See legend of Fig. 1 for further explanations about figure content

A BLAST search with contig Cg12147 in the *L. infantum* database found 84 % of sequence identity with the intergenic region between genes LinJ.14.0910 and LinJ.14.0920 (Fig. 4a). Remarkably, both genes are identical and they code for a putative calpain-like cysteine peptidase. In the *L. major* Friedlin genome there is only one annotated copy (LmjF.14.0850, Fig. 4b); however, in *L. braziliensis* and *L. mexicana*, as occurs in *L. infantum*, there are two tandemly arranged copies of this gene (Fig. 4a). When contig Cg12147 was aligned to the recently available *L. major* strain LV39c5 genome draft (TriTrypDB), it mapped into the super contig KB217907 of *L. major* LV39c5, and, interestingly, the sequence was found to be flanked by two copies, orthologous to gene LmjF.14.0850, which we have named Lv ~ LmjF.14.0850.1 and Lv ~ LmjF.14.0850.2 (Fig. 4a). Furthermore, we found that the sequences of two other *de novo* assembled contigs, Cg12142 and Cg12135, were located in the intergenic region between these two tandemly repeated genes (Fig. 4a). Hence, based on this evidence, we tested experimentally by PCR the hypothesis that LmjF.14.0850 might be also duplicated in the *L. major* Friedlin strain, and that the contigs Cg12147, Cg12142 and Cg12135 would be part of the intergenic region lost during the genome assembly (Fragment 1 and Fragment 2, Cloning strategy, Fig. 4c). The amplified region was 3.2 kb in length, and, after sequencing, it was found that it contained the three *de novo* contigs (Fig. 4c). In addition, an exact copy to gene LmjF.14.0850 was found, being named LmjF.14.0860, as this ID name is not assigned in the current *L. major* genome annotation (Fig. 4c). A plausible cause for the collapse during sequence assembly of *L. major* genome is the absolute sequence conservation existing between genes LmjF.14.0850 and LmjF.14.0860, although, they present different UTRs, especially the 3’-UTR. The differences between both genes were graphically visualized after analyzing the distribution of RNA-Seq reads on the new assembled region (Fig. 4b, c).

### A new gene (LmjF.19.0305) in the chromosome 19 of *L. major* Friedlin was uncovered after analysis of the *de novo* contigs Cg12153 and Cg12159 (region H153)

**Fig. 5:**
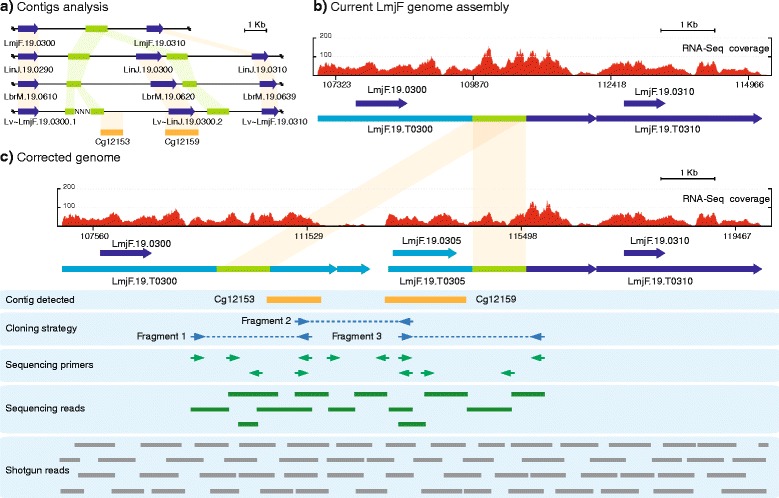
The gene LmjF.19.0305, absent in the current *L. major* Friedlin genome assembly, is flanked by a repeated element. **a** Comparison of the LmjF.19.0300-LmjF.19.0310 genomic region and its equivalent genomic loci in different *Leishmania* species (LmjF: *L. major* strain Friedlin; LinJ: *L. infantum*; LbrM: *L. braziliensis*; Lv: *L. major* strain LV39). The location of the *de novo* assembled contigs Cg12153 and Cg12159 regarding the *L. major* LV39 genomic region (contig KB217919; TriTrypDB database) are shown. The positions of the 1-kb repeated element are denoted by green boxes and the conserved areas in green shadow. **b** Distribution of RNA-Seq coverage reads and transcript definition after alignment with the currently annotated genomic region containing the genes LmjF.19.0300 and LmjF.19.0310. **c** Distribution of RNA-Seq coverage reads and transcript mapping on the corrected genomic region. See legend of Fig. 1 for further details about figure content

Clear evidence that contig Cg12159 might represent another genomic region lost during assembly of *L. major* genome was obtained after a BLAST search in the *L. infantum* genome database. It was found that contig Cg12159 had a sequence identity of 96 % to the complete CDS of gene LinJ.19.0300, a RNA-binding protein containing a zinc finger, CCCH-type motif (TritrypDB database). Analysis of other *Leishmania* genomes at TritrypDB showed the presence of orthologues to gene LinJ.19.0300 in all the other *Leishmania* species (*L. braziliensis*, *L. mexicana* and *L. tarentolae*) except for *L. major* Friedlin strain (Fig. 5a). Moreover, this analysis indicated that the genes surrounding gene LinJ.19.0300 were conserved and syntenic in all *Leishmania* species, including *L. major* LV39 strain (Fig. 5a). Thus, we hypothesized that the orthologous to gene LinJ.19.0300 might be present in this region but it was probably missed during assembly of the *L. major* (Friedlin) genome. To experimentally test this hypothesis, a comparison between the genomic region surrounding gene LinJ.19.0300 and the equivalent region in *L. major* was carried out. In *L. infantum*, the upstream gene to LinJ.19.0300 is LinJ.19.0290, whose orthologous in *L. major* is LmjF.19.0300, and the downstream gene is LinJ.19.0310, whose *L. major* orthologous is LmjF.19.0310 (Fig. 5a, b). However, when both genomic regions were aligned, a gap in the *L. major* sequence, relative to the *L. infantum* sequence, was observed. The genome draft of the close relative *L. major* strain LV39c5 was also analyzed, finding orthologous genes to LmjF.19.0300 and LmjF.19.0310 in the supercontig KB217919 (TriTrypDB database) to which we named Lv ~ LmjF.19.0300.1 and Lv ~ LmjF.19.0300.2 (Fig. 5a). Interestingly, sequences highly conserved (95 % of sequence identity) with contig Cg12159 were present in the intergenic region between these two genes. Additionally, we found that *de novo* assembled contig Cg12153 also mapped to this intergenic region (Fig. 5a).

After a detailed analysis in the different *Leishmania* genomes of the genomic region absent in the current genome assembly of *L. major* Friedlin strain (Fig. 5a, b), we found a sequence element of about 1-kb that is repeated twice (surrounding the LinJ.19.0300 gene) in all *Leishmania* species except the *L. major* Friedlin strain, in which a sole copy is mapped. Therefore, we hypothesized that the collapse during the assembly of the *L. major* Friedlin genome was caused by the presence of this repeated element. Hence, we designed oligonucleotides flanking this repeat element and within contigs Cg12159 and Cg12153 (Fig. 5c, Cloning strategy). After sequence analysis, the hypothesized gene, which was named LmjF.19.0305, was revealed together with the other 1-kb repeated element (Fig. 5c). This repeated element does not correspond to any SIDER element (either type 1 or 2) previously identified by Smith *et al.* [25] in the *L. major* genome. In summary, our results demonstrate that the existence of a repeated element surrounding gene LmjF.19.0305 led to a collapse during the genome assembly and, consequently, this gene was inadvertently excluded from the final assembly. This finding was also confirmed by the alignment of RNA-Seq and shotgun reads (Fig. 5c).

### The analysis of contig Cg12145 led to the identification of an additional 1.1-kb of DNA sequence that fits in the intergenic region between genes LmjF.36.5970 and LmjF.36.5980 (region H145)

**Fig. 6:**
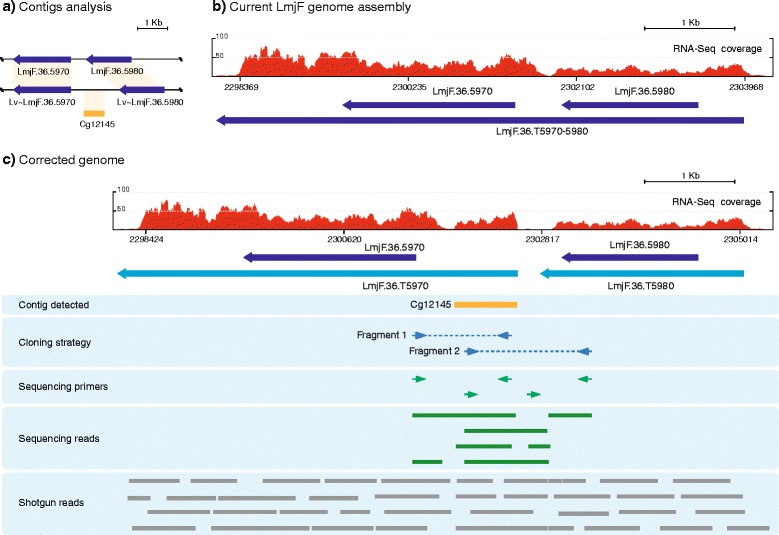
Re-construction of the *L. major* Friedlin genome at the region occupied by the genes LmjF.36.5970-5980. **a** Comparison of the LmjF.36.5970-5980 containing genes in the current genome assemblies for two different *L. major* strains (LmjF: *L. major* strain Friedlin; Lv: *L. major* strain LV39). The *de novo* assembled contig Cg12145 mapped only in the LVc39 genome. Homologues regions are indicated by orange shadow. **b** Distribution of RNA-Seq coverage reads and transcript definition after alignment with the currently annotated genomic region containing the genes LmjF.36.5970 and LmjF.36.5980. **c** Distribution of RNA-Seq coverage reads and transcript mapping on the corrected genomic region. See legend of Fig. 1 for further explanations about figure content

A clue for the location of contig Cg12145 in the *L. major* genome was obtained after searching the ongoing project of sequencing the strain LV39c5 of *L. major* (TriTrypDB database); the sequence of this contig was found to be highly conserved (99 % of sequence identity) in the supercontig KB217888. The boundaries of this sequence in KB217888 were used in BLAST searches against *L. major* Friedlin strain genome, and both ends mapped to a collinear region in chromosome 36, located between genes LmjF.36.5970 and LmjF.36.5980 (Fig. 6a, b). Moreover, we could also find in the supercontig KB217888 the orthologous to genes LmjF.36.5970 and LmjF.36.5980 (Lv ~ LmjF.36.5970 and Lv ~ LmjF.36.5980, respectively), surrounding the Cg12145 homologous sequence (Fig. 6a). These findings suggested a possible collapse in this region during assembly of the Friedlin strain genome. This hypothesis was experimentally tested by PCR amplification (Fig. 6c, Cloning strategy). After sequencing of the products, it was determined that a region of about 1.1-kb in length was lost during the *L. major* genome assembly. It should be noted that sequencing of this cloned PCR product was particularly complicated, probably due to long runs of G that may be hindering the passage of polymerases. The complete sequence was only obtained after using specific sequencing kits for G + C-rich sequences. Since no long repeated elements surround the uncovered region in the *L. major* genome, the high GC content of this genomic region and, consequently, the low quality sequencing reads could be invoked as a possible cause for excluding this region in the final assembly of *L. major* Friedlin genome. The sequence of the assembled region was further validated by aligning with RNA-Seq and shotgun reads (Sanger Institute database; see Fig. 6c). Remarkably, during *L. major* transcript annotation [9], a polycistronic transcript was described to contain both LmjF.36.5970 and LmjF.36.5980 annotated genes, as no SL insertion sites or polyadenylation sites were found for the independent genes (Fig. 6b). Interestingly, after the re-construction of this region and mapping of RNA-Seq reads, two independent transcripts (one for each of the genes) could be resolved (Fig. 6c).

### Contigs Cg12130 and Cg12138 fit in a region containing a duplication of gene LmjF.27.2030 that was missed during genome assembly of *L. major* Friedlin strain (region H130)

**Fig. 7:**
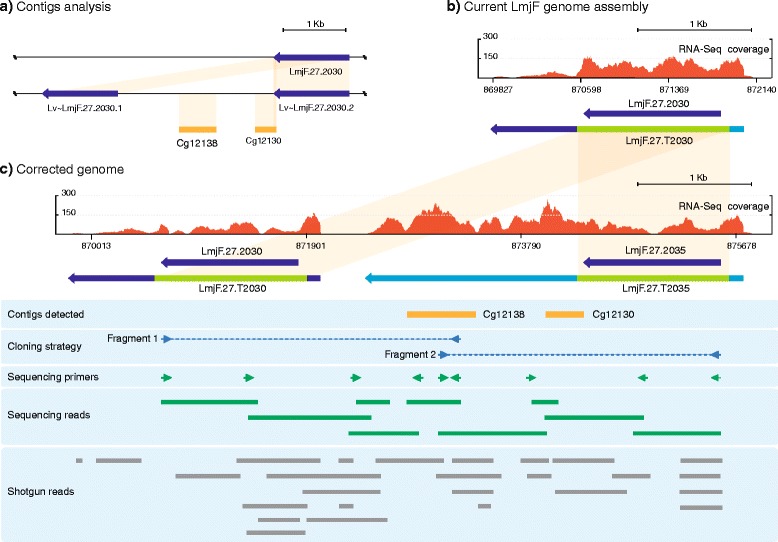
Re-construction of the locus LmjF.27.2030 shows that this gene is duplicated. **a** Alignment of the *de novo* contigs Cg12138 and Cg12130 in the supercontig KB217898 from *L. major* LV39 strain, in which two identical copies homologous to gene LmjF.27.2030 exist. Areas of high sequence identity are represented by orange shadows. **b** Distribution of RNA-Seq coverage reads and transcript definition after alignment with the currently annotated genomic region containing the gene LmjF.27.2030. **c** Distribution of RNA-Seq coverage reads and transcript mapping on the corrected genomic region. See legend of Fig. 1 for further explanations about figure content

Cg12130 was found to contain at its 3’end a stretch of 60-nucleotides identical to a sequence located just downstream of the gene LmjF.27.2030 in *L. major*, including 4 nucleotides located at the 3’ end of the CDS (Fig. 7a, b). The probable genomic location of this *de novo* contig in this locus was further suggested after the alignment of the contig to the *L. major* LV39c5 genome: it was observed an almost perfect match (99 % of sequence identity) with the supercontig KB217898. Moreover, we found the existence of two homologous copies of gene LmjF.27.2030 in the strain LV39c5 genome (Lv ~ LmjF.27.2030.1 and Lv ~ LmjF.27.2030.2), suggesting a possible collapse in this region during *L. major* Friedlin genome assembly (Fig. 7a). In addition to Cg12130, another *de novo* assembled contig (Cg12138, 602 nucleotides in length) was found to align between the duplicated genes found in *L. major* LV39c5 strain (Fig. 7a). In order to check this possibility, two pairs of oligonucleotides were designed for PCR amplification of the missing region (Fig. 7c). Positive amplification products were obtained for each PCR reaction. After cloning, sequencing and assembling an exact copy of gene LmjF.27.2030 was found; this new gene was named LmjF.27.2035 (Fig. 7c). The collapse of this region during genome assembly, induced by the existence of two identical copies of gene LmjF.27.2030, was favored by the difficulty found in sequencing the intergenic region. In fact, this missing region was not completely covered by the shotgun reads existing in the Sanger Institute databases (Fig. 7c). RNA-Seq reads alignment allowed us to validate the assembled region. Using current genome assembly, LmjF.27.2030 CDS showed 2–3 fold more RNA-Seq coverage than the 3’-UTR (Fig. 7b). Now, after reconstruction of this genomic region (Fig. 7c), LmjF.27.2030 and LmjF.27.2035 coding regions and UTRs do not show such a difference.

## Conclusions

We have identified seven regions of the *L. major* (Friedlin) genome that were lost during sequence assembly (Table 1). This led to the uncovering of six new genes (LmjF.15.1475, LmjF.15.0285, LmjF.24.0765, LmjF.14.0860, LmjF.19.0305, and LmjF.27.2035), and correction of the annotation for two others (LmjF.15.1480 and LmjF.27.2030).

Our exhaustive analysis and the results obtained support that it is unlikely that many other regions are missing in the current assembly of *L. major* genome. This assertion is based on the fact that the 15 contigs (*de novo* assembled from the non-aligned RNA-Seq reads [9]) were mapped to only seven genomic loci. If many other genomic regions remain misassembled, this remarkable concentration of *de novo* contigs would not be expected. Against this argument, it must be discussed that we have used RNA-Seq reads for the *de novo* assembly of contigs and, therefore, unexpressed genomic regions with assembly errors have not been inspected. Nevertheless, it should be taken into account that *Leishmania* has a compact genome and that the genome coverage of the RNA-Seq reads used in this study was 90.75 % [9]. Nevertheless, DNA genomic sequencing of the *L. major* Friedlin strain genome with NGS methods would be convenient for identification of any remaining misassembled regions and, more importantly, for defining the exact copy number of tandemly arranged genes, a task that could not have been performed through the classic Sanger sequencing. At this moment, our group is considering this issue.

In spite of the fact that we have documented the existence of seven misassembled regions in the current *L. major* genome assembly, it must be concluded that the Friedlin strain genome is one of the most solidly annotated genomes. Therefore, it must be recognized the great labor performed at the Sanger Institute, and the importance of maintaining, openly accessible, all *L. major* sequences in databases, including those containing unassembled shotgun reads, which have been particularly useful for this study. Nevertheless, as demonstrated in this study, the *L. major* (Friedlin) genome cannot be considered as set in stone.

## Additional files

Additional file 1:
**Oligonucleotides used for amplification and sequencing of the cloned PCR products.** (PDF 84 kb) Additional file 2:
**Nucleotide sequences of seven new assembled regions that were lost during the** ***Leishmania major*** **(Friedlin strain) genome assembly.** (PDF 96 kb)

## Abbreviations

CDS: Coding sequence
Cg: Contig
ESTs: Expressed sequence tags
NGS: Next-generation sequencing
ORF: Open reading frame
RNA-Seq: High-throughput RNA sequencing
SIDER: Short interspersed degenerated retroposons
UTR: Untranslated region.
