# Supplementary material for: Resequencing and assembly of seven complex loci to improve the Leishmania major (Friedlin strain) reference genome
Source: Parasit Vectors. 2016 Feb 8;9:74. doi: 10.1186/s13071-016-1329-4 (PMC4746890; doi:10.1186/s13071-016-1329-4)
Supplement: Additional file 1: — Oligonucleotides used for amplification and sequencing of the cloned PCR products. (PDF 84 kb) [file 13071_2016_1329_MOESM1_ESM.pdf]

Material supplementary 1

Table 1. Oligonucleotides used for amplification and sequencing of the cloned PCR products

Name (Lab code)	Genomic locus (Figure)	Sequence 5' to 3'	Purpose and PCR conditions*
12150_sara_F1 (537)	LmjF.15.1480 (Fig. 1)	GTCTCGAACAGCTCCTCGTC	PCR amplification (fragment 1), +12150_sara_R1; D-PCR; annealing: 61.3ºC
12150_sara_R1 (536)	LmjF.15.1480 (Fig. 1)	ATAGAGGCCTGTGACAGGGC	See 12150_sara_F1 (537)
12150_sara_F2 (540)	LmjF.15.1480 (Fig. 1)	ATGGTGGCGACAATTTGG	Sequencing
12150_sara_R2 (541)	LmjF.15.1480 (Fig. 1)	ACCACCCCTCGCTCCTG	Sequencing
12150_sara_R3 (546)	LmjF.15.1480 (Fig. 1)	ATCCGATCCTCGATTGTTTAA	Sequencing
12150_sara_F4 (589)	LmjF.15.1480 (Fig. 1)	AGGAGAGGGGAGTTACAGCA	Sequencing
12150_sRvs (590)	LmjF.15.1480 (Fig. 1)	GGTTTCCTGTCTTTGCATCC	Sequencing
12150_albaP1_F1 (591)	LmjF.15.1480 (Fig. 1)	CATCCATGACACAAGCGTTC	PCR amplification (fragment 2), +12150_albaP1_R1; M-PCR; annealing: 57.2ºC
12150_albaP1_R1 (592)	LmjF.15.1480 (Fig. 1)	GCTGATCAACAAGCTGGACA	See 12150_albaP1_F1 (591)
12150_albaP1_F2 (621)	LmjF.15.1480 (Fig. 1)	GGTCGGACAGAATCTCGATGG	Sequencing
12150_albaP2_F1 (593)	LmjF.15.1480 (Fig. 1)	GCTCACCGCTTTCACTTCTC	PCR amplification (fragment 3), +12150_albaP2_R1; M-PCR; annealing: 54.3ºC
12150_albaP2_R1 (594)	LmjF.15.1480 (Fig. 1)	TGTTTTTCGGCTCGTATTGG	See 12150_albaP2_F1 (593)
12150c2-F2 (625)	LmjF.15.1480 (Fig. 1)	GCGGCACTGCAAGATGAAG	Sequencing
12150c2-R2 (626)	LmjF.15.1480 (Fig. 1)	CGGTGTAGCATCTTCCTGGT	Sequencing
12155_F1 (548)	LmjF.15.0280 (Fig. 2)	TACGTGACCCCACTCACAAA	PCR amplification (fragment 1), +12155_R1; D-PCR; annealing: 57.2ºC
12155_R1 (549)	LmjF.15.0280 (Fig. 2)	TACGAGCTGCCACAAAAGTG	See 12155_F1 (548)
12155_F2 (568)	LmjF.15.0280 (Fig. 2)	TTGGTAAGCAGCAGATGAGG	Sequencing
12155_R2 (608)	LmjF.15.0280 (Fig. 2)	CTGTCGAAGGGCCAGCTT	Sequencing
12155_F3 (609)	LmjF.15.0280 (Fig. 2)	CGAAAGGGAAAGGGAGGAC	Sequencing
12141_F1 (556)	LmjF.24.0770 (Fig. 3)	GACGTCAGCCTTCTGTGAAG	PCR amplification (fragment 2), +12141_R1; D-PCR; annealing: 58.7ºC

12141_R1 (551)	LmjF.24.0770 (Fig. 3)	GTTCGTGCGTTGTTCTGTACAT	See 12141_F1 (556)
12141_F2 (550)	LmjF.24.0770 (Fig. 3)	GCATGCCTTGTAGACCCTTC	PCR amplification (fragment 1), +12141_R2; D-PCR; annealing: 59.3ºC
12141_R2 (567)	LmjF.24.0770 (Fig. 3)	GATACTGCACGCAACCATGC	See 12141_F2 (550)
12141_F3 (595)	LmjF.24.0770 (Fig. 3)	TTGTCAAAACTGCGTGTGTG	Sequencing
12141_F4 (596)	LmjF.24.0770 (Fig. 3)	CGCTTTTCATTTTGGCTGTT	Sequencing
12141_R4 (597)	LmjF.24.0770 (Fig. 3)	GATGTTTTGGTGGCTGTGC	Sequencing
12141_R5 (620)	LmjF.24.0770 (Fig. 3)	GCGACGTACAGGGAGCACTTG	Sequencing
12141_R6 (630)	LmjF.24.0770 (Fig. 3)	CCATAGCAGGGAAAGAGAG	Sequencing
12141_P1R (607)	LmjF.24.0770 (Fig. 3)	CGGGAAGACGTACAGGTTG	Sequencing
12142_F1 (557)	LmjF.14.0850 (Fig. 4)	ATGTCCGTAGCTGCCTCACG	PCR amplification (fragment 1), +12142_R1; D-PCR; annealing: 61.3ºC
12142_R1 (568)	LmjF.14.0850 (Fig. 4)	TCGGTTCTTTGCCGCTGAGG	See 12142_F1 (557)
Cg12142-FM-S (577)	LmjF.14.0850 (Fig. 4)	CCGCTCTCACGCATGGATC	Sequencing
12142_R2 (579)	LmjF.14.0850 (Fig. 4)	GTCTAACCCCCTCTCTCC	Sequencing
12142_F3 (598)	LmjF.14.0850 (Fig. 4)	TGAACCGTGTAACAGTTGTGC	Sequencing
12142_R3 (599)	LmjF.14.0850 (Fig. 4)	GCACCGCAAGCATACATAGAC	Sequencing
12142_F5 (569)	LmjF.14.0850 (Fig. 4)	CTCTCCTACTCTCTTAGACTTC	PCR amplification (fragment 2), +12142_R5; D-PCR; annealing: 58.7ºC
12142_R5 (558)	LmjF.14.0850 (Fig. 4)	TCACTCTACCAGCGACATCC	See 12142_F5 (569)
12142_F6 (600)	LmjF.14.0850 (Fig. 4)	CACGCTCCAAGATCAGAAGA	Sequencing
12142_R6 (601)	LmjF.14.0850 (Fig. 4)	ACGGCTTCAAGATCAGCTTC	Sequencing
12153_F1 (561)	LmjF.19.0300-0310 (Fig. 5)	CCTCGTCCAAGGACTGTTGT	PCR amplification (fragment 1), +12153_R1; D-PCR; annealing: 59.3ºC
12153_R1 (562)	LmjF.19.0300-0310 (Fig. 5)	GTCCCTCACTTGCTGTCCAT	See 12153_F1 (561)
12153_F2 (563)	LmjF.19.0300-0310 (Fig. 5)	GGTCGATCAGAGCAGTGACA	PCR amplification (fragment 3), +12153_R2; D-PCR; annealing: 58.5ºC
12153_R2 (564)	LmjF.19.0300-0310 (Fig. 5)	CCATCAACAACGCTGAGGAAA	See 12153_F2 (563)

12153_F3 (565)	LmjF.19.0300-0310 (Fig. 5)	GACTGCTGTTGGTGGGCCTT	PCR amplification (fragment 2), +12153_R3; D-PCR; annealing: 61.2ºC
12153_R3 (566)	LmjF.19.0300-0310 (Fig. 5)	CAGTCGCTAAGGTAGCGGC	See 12153_F3 (565)
12153_F4 (610)	LmjF.19.0300-0310 (Fig. 5)	GTGAAATGGACGGAAATGGA	Sequencing
12153_R4 (611)	LmjF.19.0300-0310 (Fig. 5)	CTTCCTCGTCCCCTTCTCC	Sequencing
12153_F5 (612)	LmjF.19.0300-0310 (Fig. 5)	GGTGTTGTCGACGTTAATGG	Sequencing
12153_R5 (613)	LmjF.19.0300-0310 (Fig. 5)	TTGATCCGAGAGGGTTGC	Sequencing
12153_F6 (614)	LmjF.19.0300-0310 (Fig. 5)	CGACAGGAGTGCAAGAAGGG	Sequencing
12153_R7 (624)	LmjF.19.0300-0310 (Fig. 5)	GTGCACCCGAGTCGTGG	Sequencing
Cg12145-R (560)	LmjF.36.5970-5980 (Fig. 6)	CTTCCTGGGAGAGACGACAG	PCR amplification (fragment 2), + Cg12145-I; D-PCR; annealing: 60.3ºC
Cg12145-M (574)	LmjF.36.5970-5980 (Fig. 6)	CTTCGTCGACGAGTTCATGCG	See Cg12145-NF (582)
Cg12145-I (575)	LmjF.36.5970-5980 (Fig. 6)	GGTCAGTGAAAGCGAAGGAG	See Cg12145-R (560)
Cg12145-IRS (581)	LmjF.36.5970-5980 (Fig. 6)	GACGAGATTGGTTTACCGC	Sequencing
Cg12145-NF (582)	LmjF.36.5970-5980 (Fig. 6)	CAACCTCCAGTTGCGCTTGC	PCR amplification (fragment 1), + Cg12145-M; D-PCR; annealing: 61.5ºC
12130_F1 (552)	LmjF.27.2030 (Fig. 7)	TCAAGCCTCGACCTTCACTG	PCR amplification (fragment 1), + 12130_R1; M-PCR; annealing: 58.5ºC
12130_R1 (553)	LmjF.27.2030 (Fig. 7)	GCTACTTCACTCCATCGTTTC	See 12130_F1 (552)
12130_F2 (583)	LmjF.27.2030 (Fig. 7)	GTAATCCTTCTCCGTCCTC	Sequencing
12130_F3 (584)	LmjF.27.2030 (Fig. 7)	GAAATGCACGTCTTCGTGG	Sequencing
12130_R4 (585)	LmjF.27.2030 (Fig. 7)	CGAAGAAGAACGGGACAG	Sequencing
12130_F5 (554)	LmjF.27.2030 (Fig. 7)	GAAGTTACAGCATCGCTTCATC	PCR amplification (fragment 2), + 12130_R5; M-PCR; annealing: 58.7ºC
12130_R5 (555)	LmjF.27.2030 (Fig. 7)	ATGTTGCTGAGCCGACTCTG	See 12130_F5 (554)
12130_F6 (576)	LmjF.27.2030 (Fig. 7)	CACGCGTGTGTGGTATG	Sequencing
12130_R7 (580)	LmjF.27.2030 (Fig. 7)	CATCAAGCCCGTGACGCTG	Sequencing

* For PCR amplifications, the following kits were used: D-PCR, Dream TaqTM Green PCR Master Mix (Fermentas); M-PCR, Maxime PCR Premix Kit (i-Taq; Intron Biotechnology).
