# Supplementary material for: Resequencing and assembly of seven complex loci to improve the Leishmania major (Friedlin strain) reference genome
Source: Parasit Vectors. 2016 Feb 8;9:74. doi: 10.1186/s13071-016-1329-4 (PMC4746890; doi:10.1186/s13071-016-1329-4)
Supplement: Additional file 2: — Nucleotide sequences of seven new assembled regions that were lost during the Leishmania major (Friedlin strain) genome assembly. (PDF 96 kb) [file 13071_2016_1329_MOESM2_ESM.pdf]

Material supplementary 2

The seven new assembled regions lost during *Leishmania major* Friedlin strain genome assembly. The sequence headers include current genome coordinates (1-based) where the new sequences must be inserted in between. Additionally, 100 nt upstream and downstream sequences are provided in red to help to localize the insertion place (these sequences are not part of the new assembled regions). CDS within the new assembled region are indicated in bold.

>H150:LmjF.15:598566-601599
caacgcacagacgcttagcattgtagccacacaccccaacaacactgcaacattccccttccagcgccc
gcattgcaccgctgctcgaaacctgctgctCATCCATGACACAAGCGTTCGCCTAGATAAGCACAGCCGC
CAACGTCAACACAGCGCCTGCACTTACCAGACACTTTCCGCACCAGGAGCAACGGCACCACCGCGCGCTA
TTACGGCATCAACGATACCACGGGACCGACGCC**TTAAACAATCGAGGATCGGATGGAGTCGCGGCGGCCA**
TCCAGGATCTCTTGCCACTTTGCGCGGTTGGAGTTTACCGTGTCCACACACCACTGCATTCCATGAAATA
GGTTGCCCACAATATCCCGGAAGAACTTGCCAGCTACGAAGTCGATGAAGCCAATCTGCCCACGCGCCAG
CTCGTTGTTCTTCGACCGGTCAAACATCGGCAGCACCTCCACGCCCTTCTCCTTCTCCATGTCACCCTGA
CGGTAGAACTCCTCCGTCACAGCCATCGCCCACATGCGCGACGTCTCGAACGGTTTCGTCACATTCGACA
CGTCACCGGCCTTGATCAGCGTTTCCATCACCAGGCGACGAGATTCCTGATCGTTAGAGTCGTAGCCGGC
CTTCGCCATATCATCGAAAACCCTCGACAAGTCCCCGTGGCGAGCCATATCAGTGGCCAGCACGCAATCG
ATCAGCGCGCGGTACGCATACGCAACGTCCTGCCCGCTCAGCCCCTCGAACACGTCCGCGGCGGGGTCGG
ACAGAATCTCGATGGCGAGGCTGCAGTGGTGCACCTCCAGCACGGAGTTGTTCCCGCTCGCGCTGGAGAG
GATGCCTAGCGGCGAGTCCGTCTTCAGGTAGAAGCTGTTGTTCACGCCCATGTGGTCAAGATCGTGCACC
AGTGCCGTCACGAGCAGCACGTAGCACTCCAGCTCTGTCAGGAGCTCCGACGCCTTGCCTGTGTACAAGT
ACGTGTGCAGCGTCTGGCACACGTCCACGACGTGGTAGAAGTTGTGGTACGGCACTCGGCGGTACCTGCG
GCGGCACTGCAAGATGAAGTTCAGCAGTGTCTGCTCACGGCAGCCAAACTTCTCGGGTAGACCACTGTTC
CATAGCAGGTTATACACAACACCGGCAGCCGCATCCATCGGCTCGCTGTACTTCTCGCGCACTTCAAACA
GGTTGAAGTCCGGTGAAGTGAAGTCATATGCGCCCCCGAAGTCGATCGACATCACTGCCTCACGCTCCTC
CGGCGTCACCGCGATCACCTTCGCACTCTTCGGAGCACGCTGCGCTGTAATGCTGTTGCGCTGCAGGTTC
AAGGTCATCGCCTCACGACCTGCGTTCACCGCGAACTCCAGCAGATGGCTGTTGTTGATCGAGATGCCCG
CAAACAGCGAGAACACTTTAAACGTCTCTTGATCAGTGGGTGTAAAGCAGGTCACGCTACCGTCGTCGCC
GAGCTTGTTGACGAGCTGCACCACGGCAAGCACCTCGCCATTCAGCATAATCGGCTCGCACAGGATGGAC
TGTGTGCGGTAGCCCAGCTGCCGGTCCACAGCGCTGTTGAAGCGCGAGTCAGCGTACGCGTCCATGATAT
TCTCGCCAACGCCGGACTCGGCAACAGTGCCGGCAATGCCTTGCCCGCAGGGAAACCGGATCTCGTTCGC
GCTGTCCGCCATCTTACTGTACAGCTCATTGCGCTCCTTATCCAGCAGAAACATCGATGACCTGTCCGCG
TTCAGCAGCTTCTTCGCGCCGTGCAGCACGTGCCGCACAATGCTGTCCACATCGCGGATGTCCGTATTCG
ACAGCAGCGCCACAACGTCCAGGATCGCGTCGCTCTTCTTCTTCTCCTTCAGCAGCGTCTCGTTGATGCG
GCAGTTGCGCAGCGACGCCGCAGCGAACATCGAGAACGTCTCGAACAGCTCCTCGTCGCGCCGTCCAAAC
ACGCGCGGAAGCCGCTGCCCGCTCTGTGTCACCATGTCCAGCTTGTTGATCAGCTGCGCAACGGCAACGA
TCGTGCCCTCGAACGCGATCGGCAGACACAAGATCGTGCGCGTGTGGTAGCCAGTCACCTTGTCCACGGA
CCGGTGGAACCGGTCGTCCTCGTACGCGTTCGGGATGTTCACCACGGCGCCAGATTCCGCAACGTGACCT
GCGATGCCCGTCCCCGCAGGCAGCACCACAACGTTGCCGTCCTCGAAGTGCGCCTCCAGGCTGCCGTCCG
CTTTCACCAGGAAGATGCTACACCGGTCGCTCTCCGTCAGCATCTTGGCCGTGTTGATGATGGACTGCAC
CAGCACCGACTCCTCCAACGTGTCTCGAGCAAGCCGTGTCGCCATGCCCACCATGGCCTCTGCCTTCCGA
GTCTCCCACCGCAGTGTATCGATGGAGGTAGCGTTCTGCAGTGTTAGTGCCGCCACTTGAAGCAGGTACC
CGAAAAACGGCGTGCTCGTGTCGATTGCGGTGCTCTCCACCTCCAGGCAGCCGACGACGTGGCTGTTGTA
ACATAGGGGGATGTACACCGTGTCGGAAATGGTAACGGTCGTCTTCGTCTTCGCACACCTGCCGATGGGG
GTCGATGTATCGATGGTTGCAACAGTGTCGTGCACTGGGTCGCATAGCACCGCGTCGTTTGTGTTGGCGT
AGTGCGTGCGCACTGAACAGCTCACAAGTTTGGAGAACTCTTCATTGATGGTGGCGACAATTTGGGCGAG
GGGGTTCTGGAAGATCAGGCACCGCTGCGCCAGTTGAGCGTACTCGTGCAGTCCCAGTTCCTGCGACTGC
TTTCGAGTGGCGCCTGCTGGGCATGCGGCGTCTGCTGCGTGGCCGGAGATGCTAGATATGCGCTGCTGCA
AATTGGCGAAGGCGGCGGCCTCCGCGTCGTTGAGCTGAACCCCGCAGCGCTTGTAACGGCTCAGAATGCA
GTCGCACAGCGTGAGTGCCTCGAGGAGATGGTCGTTGCCGCTCGGAACGGTGCTTGCACCCATCGAGGAC
GCCCTCTTGGCCGTGAAATATGCCATGGCGCTACGCTGGTTTCTCTCCGTCTCTCACTGTGATGCGGTGG
ACGTGCGCACGTGTCCAGCGCTGAGCGGGCACCCGTGAACGTGTATAGGCCACAGCTTTCCGATCCCTAT
GCTTTTTTCTATTGTGGTCGTTGAGCCGAGACAATGGTGAAACGATGACCGAAAACACGTCAAGGCCGAC
GACGACAATGAGGGTAGATTGACGCTGAGGTGAGGGACTTGAGACGGTTCTGACGTGCGCACACACACAC

ACACACACGCACGCACGCACTGGCAGACGGATGCAGAGATGCACGCACGACAGACATGACCGCATCCGAT
TTTTTGATTGGGTGCATGGATGGAAGAGCGCACAAATGGGGAAAAGAGCCGGGAAACAGCCAGGAGGGCA
CGCAGTGGCCACGGAGAGTAATCCGTGAGAAGGAGAGAGATGCATAGGGAGAGAGCAGCACGAAAGAGGC
CACGGCAGAACGCACCGTCTACTCCTGTGAATGACGCCGCTCCTTTTTTTACAGTTGCCTAATCGCCGGG
TCGCACATGGGGCAACTTTGAATGTGCCACAACTCTTGGCGGTCTCCATACAAGGGCAACGGGACGGGCC
AGCGGGAGATTCTACTCCAACGACGCGCTGGATAAGGAGCGGTGCAGAATCGCGGCAACAGATAGGCGGC
ATGGACTAGGCAGGTGTGGTGGACTTCATGATTTTGGCAGGCCAGACTAATATACAAGTAAAACCGAGAT
GGTGCCCGTGTACGCGAAAAGGGTTACAGTACGGTAAGAGGCGTGCGCAAGGGACACAGGGGTGCGACAT
ACGGGGTGGGGAGGCGGAGGCATTGCGTTGCTCGGGAAAAAGAGGGGGTGCCAAGAAAAAGAGAACGCTA
AACAAGGAGGAAAAGACGGGGAGAGGGGAGGGCGGTGGGCGACGGTGTAGTCCGCACACCGCGCATAGAT
TACATGCATGAAAGCAACCACAGGAGGAGGAGGAGGAGGAGGAGGAGGAGAAGCGAACAAGACGAAAAAA
AGTCCCGCTGGGCATGGCGGCGAAAAAGCAGGAGCGAGGGGTGGTAATGATGGCACCGAAAGGAGGTGAT
GAGGCCACGAGACCGTCCATGCATGCTTCCTCGTCCCAACTTCCTCACCTTTGGACGGACCTTTAGCACG
AACGTTGAGGAAACACCAACAGCACACAGCCCTCTCATAGCCGTATCCTCTCTGTGCCACGACCTTATCT
CGCACACGTTGCCTCGCCCTGTCCTCCCGCTCGCAGTACCCCCTCCAGAGACCACAAACGGTTTGCGCGA
GGCTTCCTCGCACATGTCACACAGCAGGCGCCAGCAGCATCTTGTGCTACACAAGTCTGCTGTTTTCCTC
GCCGAACAGAAGCGGGAGGAAGGAGGACAACGAGAGAAGAGCTGTTACATTGTTCTTCTCGACATCACAC
AACGCAAGCCTGGCTGCTCCTCCTCCCATCGCCTCTCTCTGCAGACCCAAAAAACTTCGAGAAGAAAAGA
AAAGAGGAGACACGCGAGAACGTCACAAGTGCACCAAGACACTGAGAAGGACAGGTAGTAGGTGACACAA
GAGATGGAAATGAGTCAAGGAGAGGGGAGTTACAGCATGTTCATTTCTCTGTCATGCAACATGAGGTCGA
GCCCCACTCTTGCACCGGCCCTGTCACAGGCCTCTATCGCCTGGTGCGAAGCAGCTGCAGAGACACACGT
GACCGCAACGCGCCAACTCGGCCATCTGAGGGCACAGCCTCAGCCTCGAACTCTACCCGCCACCCACCCA
CCCACCCCGCCTCACAGGTCGCCCCACAGCCGCTCACGTTGTGCCGGTCGCCACACGGTGCGATTCCATC
GCGATGGCACAGGCTCCCCACAGCAGCAGGACAGTGTGAGGGCCGGGGGTGGGATGCGTTCGAGTCACGC
CGGCACTCTGCCCATCACATGAATCGTGCGTGTGCGCTGTCGCAGGTCGCTCCGGCGCCACGCCATCCGG
CACCTGACGGCCGACATCCGCAGCGACATCTTGCTCTGTCCTCCCTGCGTTATAGGTGCTTGACCCTGTC
GCCAGGAGAAGCGGTTCCGCACTGGCAGGGATATAGGAGGGAGAGGGTGGGGGTGGGGGTGGGGGTGCGC
CGCTGAGCTTCCTCCCCAGAGACAGAGTTTGGGTGCACGAATGACCCCCCACCTGGGGGGGGGGAGAGAT
GCCACGCGCTGCGGGGTGCCCCCACTCCACTCCGTCTCACCAGGAGCACAGATGCACACACCTACGAGAG
AAAAAATGGGAGAGGGGGGAGGGAGGCGGGTGAGATTCGTGACGAACAACAAAAGACAGACACACGAAAA
AAGGGGGAGAGAAACGGATGCAAAGACAGGAAACCATCTTCGGGACACACACCGGAAAAAAAGGAAAGGC
GGATGAAGAGAGAATTAGGTGTGGTGCGTGTGTGTGCACCTGCACTGCTCACTCCTCCCCCTTCGTACCG
TTCAGCCTTCAACGGCAAACCACTCCTCCGAAAGACGAGCAGAGAGAGAGAGAGAGGTCTGTTCAGAAAA
GGGCTAATAGCGAGGAGAGAGGAGACAAGACGAGAGCCAAAAAAAAAAAACACGACTCACACGCCCACAT
GTGCCACACCTTGTCCTCAGCAGTGCGAGAGGCGCCCATCAACCAGAACAGGCCCTGGTGCAGAGAGAGG
GAGACTCACCAACACACAAACCATCCCGCGTCGCGGATACAGCGACGGGTACAAAGGGAAATGACGGCCC
GGTGCACATACTGAGGTGGGGATAAGACGCGTAGACACGCACAGCAGAGCGGCGACCAACGGCACTCATC
CGTCCATAGTGACAAGTGAGCTCACCGCTTTCACTTCTCGAAAGACATTCGCCGTGAGTACTTCCGCGCG
CTATTACGGCATCAACGATACCGCGGGACCGATGCC**TTAAACAATCGAGGGTCGGATGGAGTCGCGGCGG**
CCATCCAGGATCTCTTGCCACTTTGCGCGGTTGGAGTTTACCGTGTCCACACACCACTGCATTCCATGAA
ATAGGTTGCCCACAATATCCCGGAAGAACTTGCCAGCTACGAAGTCGATGAAGCCAATCTGCCCACGCGC
CAGCTCGTTGTTCTTCGACCGGTCAAACATCGGCAGCACCTCCACGCCCTTCTCCTTCTCCATGTCACCC
TGACGGTAGAACTCCTCCGTCACAGCCATCGCCCACATGCGCGACGTCTCGAACGGTTTCGTCACATTCG
ACACGTCACCGGCCTTGATCAGCGTTTCCATCACCAGGCGACGGTGGGTGTCGTTGTCTTTCTCAAACCC
GCTTGTCGCCAACTCTGTGAAGCGACTTAGCGCGTCAGCGTGCTTCGCCATATCAGTGGCCAGCACGCAA
TCGATCAGCGCGCGGTACGCATACGCAACGTCCTGCCCGCTCAGCCCCTCGAACACGTCCGCGGCGGGGT
CGGACAGAATCTCGATGGCGAGGCTGCAGTGGTGCACCTCCAGCACGGAGTTGTTCCCGCTCGCGCTGGA
GAGGATGCCTAGCGGCGAGTCCGTCTTCAGGTAGAAGCTGTTGTTCACGCCCATGTGGTCAAGATCGTGC
ACCAGTGCCGTCACGAGCAGCACGTAGCACTCCAGCTCTGTCAGGAGCTCCGACGCCTTGCCTGTGTACA
AGTACGTGTGCAGCGTCTGGCACACGTCCACGACGTGGTAGAAGTTGTGGTACGGCACTCGGCGGTACCT
GCGGCGGCACTGCAAGATGAAGTTCAGCAGTGTCTGCTCACGGCAGCCAAACTTCTCGGGTAGACCACTG
TTCCATAGCAGGTTATACACAACACCGGCAGCCGCATCCATCGGCTCGCTGTACTTCTCGCGCACTTCAA
ACAGGTTGAAGCCCGGTGAAGTGAAGTCATATGCGCCCCCGAAGTCGATCGACATCACTGCCTCACGCTC
CTCCGGCGTCACCGCGATCACCTTCACACTCTTCGGAGCACGCTGCGCTGTAATGCTGTTGCGCTGCAGG
CTCAAGGTCATCGCCTCACGACCTGCGTTCACCGCGAACTCCAGCAGATGGCTGTTGTTGATCGAGATGC
CCGCAAACAGCGAGAACACTTGGAACGTTTCCCGGTCCATGGGTGTAAAGCAGGTCACGCTACCGTCGTC
GCCGAGCTTGTTGACGAGCTGCACCACGGCAAGCACCTCGCCATTCAGCGTAATCGGCTCGCACAGGATG
GACTGTGTGCGGTAGCCCAGCTGCCGGTCCACAGCGCTGTTGAAGCGCGAGTCAGCGTACGCGTCCATGA
TATTCTCGCCAACGCCGGACTCGGCAACAGTGCCGGCAATGCCTTGCCCGCAGGGAAACCGGATCTCGTT
CGCGCTGTCCGCCATCTTACTGTACAGCTCATTGCGCTCCTTATCCAGCAGAAACATCGATGACCTGTCC
GCGTTCAGCAGCTTCTTCGCGCCGTGCAGCACGTGCCGCACAATGCTGTCCACATCGCGGATGTCCGTAT

TCGACAGCAGCGCCACAACGTCCAGGATCGCGTCGCTCTTCTTCTTCTCCTTCAGCAGCGTCTCGTTGAT
GCGGCAGTTGCGCAGCGACGCCGCAGCGAACATCGAGAACGTCTCGAACAGCTCCTCGTCGCGCCGTCCA
AACACGCGCGGAAGCCGCTGCCCGCTCTGTGTCACCATGTCCAGCTTGTTGATCAGCTGCGCAACGGCAA
CGATCGTGCCCTCGAACGCGATCGGCAGACACAAGATCGTGCGCGTGTGGTAGCCAGTCACCTTGTCCAC
GGACCGGTGGAACCGGTCGTCCTCGTACGCGTTCGGGATGTTCACCACGGCGCCAGATTCCGCAACGTGA
CCTGCGATGCCCGTCCCCGCAGGCAGCACCACAACGTTGCCGTCCTCGAAGTGCGCCTCCAGGCTGCCGT
CCGCTTTCACCAGGAAGATGCTACACCGGTCGCTCTCCGTCAGCGTCTTTGCCGTGTTGATGATGGACTG
CACCAGCACCGACTCCTCCAACGTGTCTCGAGCAAGCCGTGTCGCCATGCCCACCATGGCCTCGATCTTA
TTTGCTTCCCTCTTTCTGATGGAGATGTTGATGCTGTTTCGAACGGCAACGGTGACGCACCGGAGCAGAA
GCTGAGCACAGGTAGCCTTGTCCCTTGGGATGCAGGCACCCGGCACCTCCACGCAGCCAACGAGCTCGGA
CCGAAACCAGAGGGGGATGTATAGGGTGTTGGCAATCGTCAGCATGTTGCGCGCTTGCGCGCACTTGCCC
AAGGGCGTGACGTCGTCGATAAGGGCGGCAACACCGTTGACAGGGTCCCATAGGAGATTGTCGTTTGCGA
TCACCATGTACGTGCGGACATTACGCGAGCAAGTCACGGCTGAAATGCGCTCATTAATCGCCGCAAACAA
TACTGTTGGCTCCTTGGTGTTGTCGCAGCACTCCTCCACAAACGCGACAATGTCGTTCTGTGGGTTGTGC
GGCACGTCGGTTGCATCGTCAAGAATGCTCAGGAAGTCCTTCGTGGTCTGGTCGGGTGAAGCCGCGCTAT
TCGCAGCCGGCTCTTGCGCGGTATCAGGGAACTCGGTGCGCAGGGCTTGAATCGCCTTCAGCTCTGTGGA
GGAGAAGGATGTACCAGTCCGACGGTAGCGCGCCAGAATCGACTGGCAGAGTGCAACCGCCTCACATAGG
TGATTGGAGCCGGCTGCGCCGCAATACGGCGCATCGGGGGAAAAGACCGCTGAATGCATCGCTGTACGGC
GTCACTGCCAGTTGATAACAAATCGAAGACAGCGGCCAATACGAGCCGAAAAACAagagtgaacaaacga
acaacaaagaggccgcgtggcgtttgcgtgtatggggctcgcttgtgtgtgtgtgtgtgtgtgtgtgtgt
gtgtgtgtgtctgac

>H155:LmjF.15:108225-109015
cattggcgttgtatgaccttctctctcttcaccttttcatctctcccaccgtctcgctcctttgtacctc
ctcctcctcctcctcgttccttccgcaTACGTGACCCCACTCACAAACCCACTGCAGCACAGCCGCC
CTGTCGTCCTCCCTGCCCTTCGACAGACCCACTCGTCAGGCCACAACCCCCCGTATCAGGTCGCAGTAAT
CTGCCCAAGAGAAAGCCTCAAGC**ATGCGCCGTCTGTCTAGCCAGCTTATGTGCACCGCTGCCGCGGCCCG**
CTTCGCGTCCGCCGGCGGCGCGAAGAAGTACGACCTGTTCGGCTACGAGGTGGACACGAACACGGCGCCG
TGGATCGACAAGGTGAAGAAGTGCCGCTACTACGACGAGGCCGGCGAGGTGCTGGTGAACATGAACGTGA
AGAACTGCCCGCCGGATCTGGAGACGTACAACGCGACGCTGCAGAAGATCTACGAAGCGCCGAGCAAGCA
GGACAAGCCGGTGGAGAACGAGAGCAAGTTCTGCGCGATGATGGACCTGATGGAGGAGATGCAACACCGC
AACAAGGTGAAGCCGAACGAGGAGTCGTGGACGTGGGTGTTGAAGGAGTGCGTGCAGAGTGGGCAGTTCC
GCCTCGGCTACTGCGTTGCGAGGCTGATGGAGGCTGAGTTCAAGCGGGTGCCGGCAGAGCTGGTGCAGCA
GAACGAGGCGAACGCTTCGAAGGCGAAGGCGGACGGCAAGGAGCATCCGAGCACGCTGGCGCAGCAGCAG
AGCCTGTTCGACATCAAGATTCAGTAAAAGGTAGTGACGTTGGACTGATGACGGTGAATTGGTAAGCAGC
AGATGAGGGACGGTTTGGCGCCATGGAGGGTACGTGAGTGCGCTGATGTTTGGGTTTGTGCGTGTGTGCG
CATGTATGTGCGTATCGTCTCCATCCTTCTGCCCCCTTCTCTCTCTCTGTCACGCACACATGTCCACCCG
CTGACCTCTCTATACTGCCTAGGTGTGGACCCCTTTTATGCGGTGAACCTGGTAGACGATGACAACCCCA
ACCCCTTCCCCCGCGTTGCGTGTTCATACACATCCATCGCCTCCTAAACTCCTCTCGCCGAGCGGTTTTT
GAGTTGTGCGATGTGGGAGACGAGGCAATGGAGAGGCGGAAGCGACGGGGTGCTGTGGGAGGAGAGCGAG
GGAGAGTGCGCGCACACACGCGTGTGAGCACTACAAGCCGCTCGTCCCTCCGTCTGTGTGGATGACGTGC
GTGCAGGAAGTGTACGTGACCTTGGTTTTGTCTGTAGGAAGGCACGTGCGACCTCGCATCGATGACGACG
ACGCCGGGCGCACTGAAGCGAAAGGGAAAGGGAGGACATGACAGGGGGAGAGGGATAGCAAGTGATGAGG
GTAATGAATGGAGCGGGTGGGGGACGTTAGTGTCTCTCTCTCTCTCTGAACGCTCGTGTGGAAAACGTG
ACAGGGGCAAGTTAGCAGGGCCAACGACTCCGAGATGCGTGCCTCTGCTACTCCCTCACCACTTACGCAC
CCCCACCACACACACTCTCTTGCTCACCTCATCGCTCTTGCACACGCGCACACACCACAGGCACACGCAT
CCATACACAGCACGAGTGCTTGCACTGGCGTGCAAGTCTCTGTGTGTGTGCGCGCGCGTGCACCCCATCA
CGCTGCCTTTCTCTCCTTTTCTTTTCTTAGTCCCCTATTGTTTTGTTCTGTAGGCATAATGAATGGAGTG
TTAGTTACACCAACGCGAAACGACAGGGTCGGCAGCCACGTCCATGGAGTCGAGGCGGCGCGCACCCGAC
AGGAAAGGGAGAGGAGGAGGGAAATGCATGCGTAGGAAGGGCGAATGTCGGCGTCCATACCTTCAGTCCT
TCCCACCTCGCACCGCTCTCCCGCCCGCCCCCACCTCCTCCGCCCTGCTCTGTGTGCCGGTGATGGTGAT
CAACGCGAAAGGGAGAAAAAGTGTGCGAAACAGAAAAGATGGAGTGGTGTCAAGACGCCTTTATGAGTTT
TCCCCCTCGTCTCGCCCTTCTACCGCTGCGCTGAGCACCGCAGTGGCGAAAAAGAAGAGGAGGGGGAGGG
GGAGAGCTCACAGCACTTTACGGTCTCTTGGGTGCGCTCTCTCTCTCTCTCTCTCTTTCACGCTCTTCCT
CTCTCGATGTGTGTGTGTGTGTGTGTGTGCCGGAGCGCCATCGCGCGTCTACGATTCTCTCTCTCCCTCTCG
CCCCTCCCTCTACCCTTCTTTCATTCGGCGCACACTGCGCGCCGTTCCTACACGCAGTCCCGCACAAAGA
CTTCCCAACAACACTCTCGTCCCCAGCAAAGCTGGCCCTTCGACAGACCCACTCGTCAGGCCACAACCCC
CCGTATCAGGTCGCAGTAATCTGCCCAAGAGAAAGCCTCAAGC**ATGCGCCGTCTGTCTAGCCAGCTTATG**
TGCACCGCTGCCGCGGCCCGCTTCGCGTCCGCCGGCGGCGCGAAGAAGTACGACCTGTTCGGCTACGAGG
TGGACACGAACACGGCGCCGTGGATCGACAAGGTGAAGAAGTGCCGCTACTACGACGAGGCCGGCGAGGT

GCTGGTGAACATGAACGTGAAGAACTGCCCGCCGGATCTGGAGACGTACAACGCGACGCTGCAGAAGATC
TACGAAGCGCCGAGCAAGCAGGACAAGCCGGTGGAGAACGAGAGCAAGTTCTGCGCGATGATGGACCTGA
TGGAGGAGATGCAACACCGCAACAAGGTGAAGCCGAACGAGGAGTCGTGGACGTGGGTGTTGAAGGAGTG
CGTGCAGAGTGGGCAGTTCCGCCTCGGCTACTGCGTTGCGAGGCTGATGGAGGCTGAGTTCAAGCGGGTG
CCGGCAGAGCTGGTGCAGCAGAACGAGGCGAACGCTTCGAAGGCGAAGGCGGACGGCAAGGAGCATCCGA
GCACGCTGGCGCAGCAGCAGAGCCTGTTCGACATCAAGATTCAGTAAGACGCTGCGTGTGCCGCTTATCA
CCTGCACTTTCTCCCACGCCTCCCTCCCCCCCCCCCCCCACACACACACACTTTTGTGGCAGCTCGTAg
acggttcggtgcagaggatgcatcgcatccgcggctgtgcgaagcgaattttgatcgtatcgttaaggaa
tgggtgagaggagatgcaagggctgcatg

>H141:LmjF.24:268817-269759
gttcgttgttgtcagcttgtgcttctctcggtcctctcctctcgcgcggcatcacgtgtgcacgcctcct
gagcatctctcgctcgctctctcttcctccGCATGCCTTGTAGACCCTTCATGTCCAACGCGGTGCACGG
TGCAGGTTGCAGCGCACATCGCCTCCCCCCCCCAAAGCGTTTACGTGTATGCGCGGTCTCGCCCCACCCC
ACCCCCGCTCGCTTCCCGCGGCGCGTCGCTTCATTTTTTTTCTCTTTATTGTCAAAACTGCGTGTGTGTT
TAGAGGTGGGGGGGGGGGGTAGCCAAGCCGCCCCCACCACCTCTATCCACGATAGCCCTGCCAATGCCGA
ACCACCTCTGGAGGTGGCAGGGCCAAGTGCCCACGGCGCAGGGGGAGGGGGGGTCAGAGCGGTTTGATCG
CTGCTGATGTTGGCCGTCAGGTTATGCATGGCGTTGCGTCGGAGAGACCCGCGATCGTGAACGTATTTGT
ATCACCCGTGTGATGGGCAACGTGCCAACGTATGTGACCCGATTGCACCCCACACCCGCCCCACGCTGCT
TCGCGCCACCCAGATGGGGAATGCACCAGGTGGTGACCGACATAATGGAAGCTGGTGTGGATGGGTGGAG
CTCGAGGCAGAGGTCGTGCTGAGATGGCTGAATGTCCGTGCATCACCGCAATGCAAGTGTCCCGCGCTGC
TTCGCGCCATGCGGATGGGGACCCCTGTGAAAGGGCTGGCGGGGCGCAGGTAGACTGGGATTGAACACAT
GTTGTGCGTGGCAGAGAAGTGCGCACGCGTTGTGGAAGACAGGCAGGCAGAGGCAGTGAGACGCAAGCCT
GCTCGTTCGACTTGCCGCACCCCTCCTCTCCTCCTCCTTAGGGGCCATCTCCGCCGTCCCCAGAGCGAAA
GAAACGGGCCGCCCCTCCCTGCATTCCACACTGACTCTCGAACTCGCTCCCTCCCACCCCACCCTACCGC
TTTACTTTCATATGCGCCTCCGCCATTCACCGCAACCAACACAAACCTCTCATTTCGACTCGCGTGCGGG
CTCATCAAGCATTACTCGCACCCACACACACACCCTCTATTCCTCCACCGCCTCTCTCCTCCTCCCTCTG
CCCCCGCACACACGGTTAATGCGAAGCGCCAACTAGA**ATGTCCCTCCAAAACGATCTGCAGACCAAGGCG**
GCGTCGCACGTGCGCTCGCGCGGTGTATATGTCCTGACGAACCGCTACCTGAACCGCGGTACGGCCTTCA
CGACAGAGCAGCGTAGGAAGCTTGGCATCCTTGGTGCCCTGCCGCCCACCGTCGAGACCCTCGAGATGCA
GGTCGAGCGTGCCTGGACGCAGCTGTGCCGCTACGACAAGCCCATCAACCGCTACCACCACCTGGTCAGC
ATCCATGCCACGAACACGACGCTTTACTACGCCCTCGTGCTCACCCACATCGAGGAATTGCTCCCCATCA
TCTACACGCCGACCGTCGGCGAGGCCTGCCAGAACTTCAGCAACTACTGGGTGCGTGAGCGCGGCATGTT
TTTGAGCAAGTACCTCAAGGGTCACTTCTCTGAGGTGATAAAAGATTCCCTGTACGACAACGTCGACGTC
ATCGTGATCACCGATGGCTCCCGCATTCTCGGCCTGGGCGACCTCGGCGCCAACGGCATCGGCATCAGCA
TCGGCAAGTGCTCCCTGTACGTCGCCGGCGCCGGCCTGCATCCGAGCCGTGTGCTGCCGGTCGTCATGGA
CGTTGGCACAAACGCGGAACGGTATCTGAACGATCGCGAGTACCTCGGCACCCGCGAGAAGCGCCTCGAT
GACGATCAGTTCTACAGCCTGCTAGACGAGTTCATGGAAGCTGTGAAGGACACCTGGCCCTCCGCTGTCG
TGCAGTTCGAGGACTTCAGTAACAACCACTGCTTCGACATGCTGGAGCGCTACCAAAAGAAGTACCGCTG
CTTCAACGACGACATCCAGGGCACCGGCGCCGTCATAGCTGCTGGTTTTCTGAACGCTGTCAAGAAGTCC
GGCCTTGGCCCTCTGGAACAGCGCATCGTCGTCTTCGGCGCCGGCTCTGCTGCTGTCGGTGTGGCGAAAA
ACATCGCCCAGCTGGCCTCTCGCATGTACAACATAGACGTTGCTGAGGTGCTGAAGACGTTCTACCTGGT
CGACACGAAGGGCTTGGTGACTGAAACCCGCGGTGACAAGCTGGCGGCGCACAAGGCGCTGCTGTCGCGC
AAGGACATCTCGGCCGAAGACAGCCAGAACCTGAAGTCGCTCGAGGATGTCGTGCAGTTCGTGAAGCCGA
CAGCCCTGCTTGGCTTGGGCGGTGTCGGTCCTGTGTTCACGGAAGCGATAGTGAAGAGCGTCGCCGCCAA
CGCTGCCCGCCCGATCATCTTTCCGCTGTCGAACCCCACGAGCAAGTCGGAGGTTATGCCAGATGAGGCG
TACCGCTGGACCAACGGTGCAGCTATCATCGCCTCCGGCAGCCCCTTTCCGGCCTCCACCATCAACGGCA
AGACCATCAAGCCGTCGCAGGGCAACAACCTGTACGTCTTCCCGGGTGTCGGCCTTGGCTGCGCGCTTGT
ACAGCCGTCCTATATCCCGGATGACCTCCTGGTGGCTGCCTCGGCGTGCCTGAACCTGCTCACGACGCCG
GAGCAGATGGAGGCGGAGCAGCTGTACCCGCCGTTGGAGGACATTCATAACATCTCTGCCCACATCGCCA
CCGAGGTTACCATGGAGGCGCAGCGCCTCGGCATCGACGGAAACAAGGGCCTGCCGCGCGAGAAGGATGC
CATTCTGGCGGTCATAAAGGCATCTCAGTGGGTGCCGCACTACCCGGCAGAGCTGGACGTCAGCCTTCTG
TGAAGGAAGGCGGCTAGACTTCTGCACTGTGCGACGGTAGCGCTGCGGGAAGGCGGTGCCGCTGTTTGCA
TACGTGCATATGTGCTGTGTGTTACTGTGTAGTTGTATCCGCCGTGTGCACGCGCATCTGTCGCTCAACG
CTACCTATAAGCCCTTCTTTCTTCTTCATGCCCCGCACTCCGGCCTCACCTCCCTCCCTATCGGCCCCCT
CCATGAACGCATGGTTGCGTGCAGTATCTTCTTCTGCGCGCCTCTGTGCCGATGCTGCCCTTCCTGCACA
TGGTCGTGTGCGTGGGCGTGTTTGTCACTCTTCGGAGTTCTCCTTTACCGTTATCCCCCACCCCCTCCCC
CGCCTCCTTTTTGTTTGTACTTGTTGCATTGCTTCCATTTCTATTATTATTATTATTTGAGACGGCGATG
CGCGCGTGCGTATGTGTGTACTTATGTGTGTATCCGCCTTCTCTTCCTTTGAGCCCCGACTTCACTCATT
CCTCTTTTCGCTTTTCATTTTGGCTGTTCTGCGGATCGCCCCCCCCCTCTTCATCTCCCTCTCCCTCTT

TCGACCCCACCCCCCTGTGACCCCCCCCTTGACTCCCTTCACTGGGTACCGCCTCCGTCCTGTTGTGGCT
GTGTGCCTCCTAGCATGCGTGCGTTTTCTTCGTGGGGGGAGGGGGTATCCGCTTGCGGATGTACCGATGC
TCATGCTGACGCGTTCCCTTACTTCGCGCGTGCGTGATTTGTTTGTTTGTTTGTTTTGTTGGGGGAGGGA
GGGGGACGGATCGTCACTCCACATGGTGTTTGCCCGTGGGCGTCTCTCACTTCCTATCACTCTGTGTGTG
TTGGCCCCCTGCCGGATGTCTCTTTCGGGTGTGCGTGTGCATCTCGCTTCTTCCGCGCGAGGATGTCGCA
CTCGCTTGCCATGCCGTGTCTTGAGCGTCCCTCAACAAGCTCATCTCTCCCCTTTCCACGCCTCCTACAT
GCTGGCGTCACCTTTTCGCCCCGGCACACCGGAGACGACCCCTTCCCCCTTTCCCACCCTCTTCCCGTAT
CCCGGCTGTCGACAAACTGAGGGGACGTGAGTGGTGAGGTGGAGAGATAGCAATAGGCCGACAGACAGCG
TGGGGAGGGGAGTCACGCACTAGCGCGAGAGACAGGCAGAGGGGAAGAGGGGGCAACCAGAAAAGAAGCA
ACAACGAAAGCTAACACAAGAGTGATCGGGTATCTTTTTTGGGCGCTTAGGGGGCAATGAAGGGCGGGGC
AAGGGGGTGGTGAAGGTGTGGATGAGGGAGTGTGGTCATGCGCATCGCCCACAGGAACACGCTCCCTCCT
CTCCCCTCTGCCCCTCCCTCCTCTCTCTCTTTCCCTGCTATGGTTACTGTCTCACGCAGGCCCGGGGTGT
CGCTCTCCTTCTTGTCGCGATGGAGCCTGGCACTTACTTCTTTGTTGAAATATGTTTCTGTTTTCTGTTA
CTTCCGACTTTTTGCGTGCGCGTGTACCCTCTTTCCTCGCATCCGGTGCGTGGAAACTCAGAGCTCTGCA
CCCCTCCCCACTTCCCACTCTCTCTCTGCCTGTGTGTGTTTAGAGGTGGGGGGGGGGTAGCCAAGCCGCC
CCCACCACCTCTATCCACGATAGCCCTGCCAATGCCGAACCACCTCTGGAGGTGGCAGGGCCAAGTGCCC
ACGGCGCAGGGGGAGGGGGGGTCAGAGCGGTTTGATCGCTGCTGATGTTGGCCGTCAGGTTATGCATGGC
GCTGCGTCGGAGAGACCCGCGATCGTGAACGTATTTGTATCACCCGTGTGATGGGCAACGTGCCAACGTA
TGTGACCCGATTGCACCCCACACCCGCCCCACGCTGCTTCGCGCCACCCAGATGGGGAATGCACCAGGTG
GTGACCGACATAATGGAAGCTGGTGTGGATGGGTGGAGCTCGAGGCAGAGGTCGTGCTGAGATGGCTGAA
TGTCCGTGCATCACCGCAATGCAAGTGTCCCGCGCTGCTTCGCGCCACGCGGATGGGGACCCCTGTGAAA
GGGCTGGCAGGGCGCAGGTAGACTGGGATTGAACACATGTTGTGCGTGGCAGAGAAGTGCGCACGTTGAA
GCGCACGCTACGCCGGTGTTCTTTCTGGGCTCACAGACGGAAACTAAAGGGGAGGAACTACAGAGGAGGC
AATGCGCGCGAATGCTTCCGCGGCAGCGTCGTTGCCACCATCATCATCACCCGTTCCCCCCCCCCGCCTC
GTCCCCATCTGCGGTGTCACCGCACAGCCACCAAAACATCGACAACTAAAGTACTTTATTGCTTGTTGCT
CTTCTTCATGTACAGAACAACGCACGAACgtatgcatacaaggattgttgaaggaaaaagagggagacgt
gtcatccgcatccctcgctcttccccctgcattcctcttcattccctcataccccctccc

>H147:LmjF.14:341556-342022
tcaacgaaaggagaggaggacagaacgaagtcgatgagcaccgcgcacacaaacaccatgcgaacgccac
tacgcacacgcccttaccgcctctctctacATGTCCGTAGCTGCCTCACGTTGCCAGCTCGAAGGTGCCC
CC**TCACGCCTTCGCAATCGGGTTGGCCTCGAAGCTGATCTTGAAGCCGTTCGGCTCGCCCTCGATGAACA**
TCTCCGTCGTCGTCGGCGCAATCTCCAGCTCACACTTGAACTCGCCAGACTCCTCGTCTTTTTGCATTGT
CGTGTTGCCGATGGCCTCGATCTTGCTGTCCTTGCCGAACGCGACCTTCACCACCATTTGGTAGTTCGTG
GTGTCGTTGTAAAACGCCCACTTGCGCTCCTCGTCGTTCACGATGCGGAACAGCAGCCCGTTGCCGTTGT
CCTTGAAGCACTTCACCACGGTGTTGCCGCTGTAGCCGGGCTGGCCGTTCTCGTATTTGATCTCAGACAT
GATGGTGGGTAGGGGCGTGGGATAGGAGGGGGAGGGGGCGATGAGGAGAGGGAGGGCGATGGGGGTGTGT
GGCGGGGATGTCGCTGGTAGCGTGAACGTGTACAGTTGTGCTTGCTTTGTGAAGTATGCAGAGGAGAAGG
CTTATGCTAGCTGATGATGGTGTCTGGGCAGAGACACAGACACACGAAAAGGGAGAGAGGGGGTTAGACG
CTCACGTGCGCATGCAGGGCATCGGCGGACCAGCGGAGACGAAAAAGAAAGACGAAGGGGAGGGGAGAGA
GAGGAGTGCATGTGTGTGAATGTTCTCAAAGTGCGTCGCGCCTGAGTGGCGTGCTACCGCATGCTGACCC
GCGCGCGCTGTACAGTAAGCAGGAGCATGGAATGGTAGCAGTAGTCCTCGCTGCCCCTAGTCCTTCGTAG
AGCGACGCGGCAGGGCATCGGCGGAGGCCTGGCTCAAGCAAGCCTTCAGAGGGGCCGGCAACCGCGCGTG
GGCCGCGCCGGCACGCACACCGCCACCTAAATCCGGCGAAGACGACCCACAAAGAGAGAGAAAGAGCACG
CCGTGCTGCAAGCGCGCTCTTCCCTCGATGCTTGTTCCGCTCCTGCCTGCGAGGTCGCTCTGGGTCTTTG
TTCGTGGGCTTTGCCGCATGTTCTGTCTCTACATGTCGTGTCGACGAGGGGAGGGAGGGAGGGAGGTTGA
GAAAAAGGTGAAAGGCGTCCGCGGGGGACCGCACGAGATAAGGGCGAGGGGAGGGGGCAGGGCGCAGAAC
AGGTGTCTATGTATGCTTGCGGTGCATAATGTTCGTGGGCGAATGCACGCGAGAGCGAGAAATAGAAACA
CGGAGGGAGTGAGGGGATATCAGAGGCGAGGAATGCGATCCATGCGTGAGAGCGGACCAAGTCCGGTCCT
GTCTCTCTCTCTCTCTTTCATACACACATACATCATCATCTCCCTAATGCACACATCCACACATGCTAGC
AACGCGGGGCATTGACGGGCATGCCTCAACCCCATGGGGATCGCATCATGCACTGTCACCCGCCCGCCCG
CACGCACCACCCCCACCACCCTCTCCTACTCTCTTAGACTTCACTAACCCCCCTTTTCCTCCTCCAGAGT
TTGGAAGATCGATCCTCTCTCTCGCCCCTTCCTACCCACCATAGTCCACTTGTTTTCTCACTTCTCACTT
TGTTTTCCATAACGCGTGCGTGCTCGTTGTTCGAAACAGTTTCCCCAGGCCTTCGCTCCGTTCCGTTCTT
TTCTCTCGTGCACACCGCAAAATACACAACAGCCACCTCAGCGGCAAAGAACCGAACACGAAACGAAGAC
ATGCGCGTCCGCACGTGTCGTCGGTGCATCTGCCGGAGAATCTGTGCGACACACAACGCGAGAAGACGAA
AGGGCGTGAGGAGGAAGAGCGAAAAAGAAGAAGTAGGGTGAGAGGGAGGCGATCACCGGAACAGCGTACA
ACCGAGACGATGAAGGCGTGCGGCGACGGCGGAGGGCAGAAGGGCAGGGAGGGAGGGGGGACACGCTCCA
AGATCAGAAGAAGCCCATACCCACCCACCCACACTCATCCATCACTACCGCCACCACCACAATCCTGCCT
TTAAAAGGAAAAAGGTAAATAAACGATGAAGCGCGGACAACATGGCAGCGAAGACGTGGCTTCGAGAGAG

CGAGTGCGGGGCACACAGACGCATACACATAATATATATATATATACAGGCGAGAGAGGAGACGAGTAGA
GAAAGACAAGGACACATGGCGCATACGAGGCATCACAAACACCACACACACACAACAACCACAACAACGA
AACGAGACGAAAACGATGCGAGGTAGTGAAAGAATGATCAAAGATAAAAATGCGAAGAGAAAGATGGGGA
GAGCACTCCGCGCCCTATCGATGCGTCCATCACACCTCTCCACCCTCGCACTCTCTATCACAGCGACGCG
TAACAGAAATGTATCCGAGAGGCTAAAAGAGTGGAGAGTCAGGCCGTACCGCCAGGAACGTGCGTTTCGC
GAGACACGCTTGTCACTGGAGCACGTCCATGGTGAGGATGACCGACAGGGGAGCAAGAGGGCGAGAGGTA
CAGGGGGTATCGTAAGACAGAGAGACGGCGTCGAACACTGCGGAGACTGGAATCCCTGTTGCTGCGGCGC
ACTTGACAAGAGCACGCACTGCATATCTTTGCCACAGCTGCCACCCATCTTCTCTCTTCTTCTCACACGT
CACGCTACCCCTCCCTCTACTTTCTCTTTCTCGCTTGCGAGGTGCTTTAGCTTCTACATGGCATCGCTGT
TTACGTGCGAGTGCGCACACGCCCTTACCGCCTCTCTCTACATGTCCGTAGCTGCCTCACGTTGCCAGCT
CGAAGGTGCCCCC**TCACGCCTTCGCAATCGGGTTGGCCTCGAAGCTGATCTTGAAGCCGTTCGGCTCGCC**
CTCGATGAACATCTCCGTCGTCGTCGGCGCAATCTCCAGCTCACACTTGAACTCGCCAGACTCCTCGTCT
TTTTGCATTGTCGTGTTGCCGATGGCCTCGATCTTGCTGTCCTTGCCGAACGCGACCTTCACCACCATTT
GGTAGTTCGTGGTGTCGTTGTAAAACGCCCACTTGCGCTCCTCGTCGTTCACGATGCGGAACAGCAGCCC
GTTGCCGTTGTCCTTGAAGCACTTCACCACGGTGTTGCCGCTGTAGCCGGGCTGGCCGTTCTCGTATTTG
ATCTCAGACATGATGGTGGGTAGGGGCGTGGGATAGGAGGGGGAGGGGGCGATGAGGAGAGGGAGGGCGA
TGGGGGTGTGTGGCGGggatgtcgctggtagagtgaaaatcctgaggaagcttcacgatgcgtgttgtgg
tgcgtgcgtgttgttcagagcacaaacgcgtacgaaggggagcagg

>H153:LmjF.19:109375-111190
gcacttcttgccgatggggttggcgggatgaggggtggggcggggtgttgagaggaaggctcacgggctg
gggtgagtcggtgactcggcaaagacgacgCCTCGTCCAAGGACTGTTGTTAGCACACACCCTTCTTCCC
CATTCGGCTCCCCGGCAAGCGACTCTTTTTTCCGGGGTTGTCTCTTCTCCGAGTTGCGAGACAACAAGAG
GACGTCCTTGCCTAGGCGCGTGCGGGCACCTGTCCGCACTTTGGATGCCTGCAGCCACGTGTGACGAGCA
TCGACAGCGTGCAGTGCTAATGGCGTACTGCCCACTGCTGCGCTCGCGTTGTTGTGCCCTCGCCCCCGCC
GGCGCCGCTGCCCTCCTGCCTGCGCCTGCTGCGAGGGCGTCCGGCTCGGCGTTGTGCTCTATATGCCGCG
TGTAACCCGTAGATCGCTCACGGCTCAAAGCGCAACTTCTTGCACGCCATAATCTTGTCCACAGCGGCCT
GATTGTGCACACCGCGCTCGCGCTGCGTGGGCTCCTCACTTGTCAGGTGTTCATGGCTGCACACCCTCTC
TGCCCTCTCAGAGGGCCAGACCACGGGCTCTCTGTGCGCGGGCGCATGCCTATGCACGGGCCCCTTCGGC
TGGTCACACCGACTCCTGTGGGGGGCGTGCGTATTCGGTAGAGGATGGTGTGTCCCCTGCCGCCACAGAC
TCATGGCCGCGTTGCGCCTGTCGTGGCGCGTGCGGGCAAGCCCCGCAGACGCGCCCCGGTGAAATGGACG
GAAATGGAGGATGAAGACAGCGAGCCCCCGAGACAGCAACGCCGTCCTAAAGCAGGCCCCATGATCCGAA
AACGTGAGCGCTGTGTGCACGCGCTCTCAAGCACAGGTGGCATGGTGTGTCGCATTCACGCAAGGCATCC
AGAGGCCGGCAGAGCAAGAGTGATGGACGGAGAAGGCGGGAAGGGATCCATGCAAATGGCCCCCCACCCC
GCCACACACACACACACACACACAATGCATGCGTGCGAAGCATGTGCGGCATGCCCTCCATCTGACCGTG
TGGGCTGGCGCGGCGCCGATGCGGGAAGCACGCCGAGAACACGGCGAGTCGCGCCATCGAGTCCTCCCGG
CTCGAATGTGGCGAGCCGCTGCAGAATCTTGTGGAATGCCGCGGCTTGAGGCGACGGAGGGCGAAGCATG
GCATGCGGCCCGGGCACAGGGACTCGGAGGGCCCTGGCCCGCAGCTGGCCAGCTTCCTGCTTGAGCTCAC
CAGGCATGCACCTTGACCACCACCTGTTATCCTCACAGCGGATGCCACCGTTTCCGGCCTGGGGCAGGAC
CCTGGGCGCAACCCTCTCGGATCAAGGCATGCCGTCCTCCCGCGTCTCCGCCTGGCGCTGATCGCGCAGC
GCGTATCGGAGAAGGGGACGAGGAAGGGCGAGAAGAGTCCGTACGACGCACGGCCCGATTCGACGGCAGG
GACGGCTACGTCCTGGTCCACCAGGAGGTAGCTGTGGTGCAGCAGAGGTAGGTGAGCTGCGGTTGTGTCT
GCGTGTGTACGTTTATTAACTTGTGCGTACTGGCATGACATGTAAAGAGAAAAGGGGGCCCCGGTGCCTT
CCACGCCAATGAATATGAGCGGGCTGATGACTGTTACTGTCAAACCAATACTCTTTGTGCGGTGCTTCGC
ACCTGGAAAACATGTCGGTGTGTTTTCATGGGACGTCGCTGGATGTCTCGCTGCTCCTCGCTTTACCTCT
CGGGGGGAGGGGAGGGGTGCAACTGCGGCGTGCGTGTGTGTGGTAGTGGTGTTCGCTTTTGTTGTCTTCT
TTTGGCGTAGTGCCGTTGTGTCGTCACGCGCCGCGACTCGGTCGTGCGGACGTCTTTTTTTTTTCGTTTT
CGTTGGTTTCTTTGTCTGTGCTGGCGTGTGTGGTACGTAGGCGTGCGGGCGCACGTCCGTGAGTTGTCCG
GGACGCGTTACCTTGATGGTTGTTGATGTGCACATGTATGTGGTCTTGTGGCATGTGTGTGTGTGTGACT
GCTGTTGGTGGGCCTTGTTGTTTGCTGGCGGGTTTCGTTCGTCTTTGCTCTGCTCTCTCTCTCCGTCCTG
CGCTGAGTATGTGTGAGTGGGGCGAGCGAGAGTGGAAGTGAGAAGGCGAAAGCGATAAGCACCGGAGGCG
TCCTGTTTCTCTGTCTGTGTCTGTCTGTCCATCTTTTCGAATGCGCGTGCGCGTGTGCGATTTGCCGACT
TGGTGGTCTTTCCGTTTTTCGATCCCAAATAACTACCGCAGAAAAGCACGCAAATGCGAGATTAGCGCAC
GGTGTTCACATGGACAGCAAGTGAGGGACAAGCACAGACGCCGACGAGCGCTCCGGGGGAGCACCTCGTA
TAAACTCATGCACGGCTGCACGATGAGTTCGACGTGCTTGCACCCACCCACACTCGCCAACGCGGGCACG
AGTGCTACTCGGTGTTTATGTGAGTCTACAGGCAAGATACTTCTCTCCACGGTTGCGCGTCTTTTTCCGC
TTGTCTTGCCACGTTGAGTGCGAGTGAAGGGGAAGAAGAGGCGCCGGAAAAGTGAGTCGGAGCTGTGGCA
GGCTCTGTTCCCTGTGAAAGCCTTCGACAGGAGTGCAAGAAGGGGGGGGGGGAATGATCGAGACAGAAGA
CAAGACGGAACCGCGTGAGGAGGAGGAGGAGTGCGTGAACCTACTCGCCGCCATTCGAATGGCACGCTGC
AACAACCATCGGATCGCCTCCTTTACCCCCCCCCTCCCCCCTCTCCCGTCCTGTTCGTCTACACGGCATT

TTCGCTATCGAGGAAGTCGGTCATGGCAAGCATTTGCTTTTTCCGGTTCACCTACCCTTCACGGTGCGCA
CGCGCGCGCACACACGCGTGCTCCCATGATCGGTCAGAGCGACTGTGTACGCGAGCGGCCACGTATCACC
ACCACCGCCTCTCCATTTTGACCTGCTATCTCTCGACTTGCGTTGTTCCTGTTTTGCACTACACACACAT
GCCCATTCCCCCCTCCACACACCAACCGGGGCCTTCTCTCTCTCTCTCACGCACACACACACACACACAC
ACACACACACACACACTTCCACACACTTCCAAACACACACACACACACACACACACTTCCACGCACACAC
TTCCACACACAGCGGAGGCGGGCGGACAGACACGTTTCGCTGTTCTTCGCGCGTGCGCGCCTTTCCTGCC
TCGCCTCTTCCCTCCTCTCTTACTGACGAGGGGACAAACATGTCGAGTCTTCGACGGCACCAATCAGGGG
CAACAGCGAACCGACGCGCGCGCTTAGAGAGGTACATAAGAGGGCTGAATCGTCAGACACACCTACACGC
ATCAAGGCGCTCCCCCTTTCCGAGCCATCACCTCACCTGCGCCAAACTCCTGCATCGCGCACGCGCTCTC
TGTCTCTCGCTACCGCGCTCTGAATTGTCTCGCGTGCGTGTTCGTTTGGCAGTTCATTGCTCGCGCAAGT
TTGTGATGACAAGCACCCGCCCCCTTCCCCCTTCTCCCCTCACTCCAGGCCCATACCCTCGGTGCCTCGG
GTGTCCTTTTTTGTCCATGCATCCCTCTCTCTCTCTGTATTCCCTCTCTCCTACGGACTCGGGCCCGCTC
GCCTGTTCGCAACATCTTCTCTCTTCTCGCATTTTCGAGAGCAACGACACCCCCCTCCCCCGACCCACCC
ACTCACTCCCCGCCCACACTTCCTCGTGTGGCGCAGTAAAGAAGACCACGACTCGGGTGCACACCGCCGC
CTCCTCTCTCCACTCCTCTTCTACTTGAACACGAACACAGAGTGCCTTCAAGCGGAGAACAGCA**ACAACA**
CGATGAACGCCATGTCGCCGTTTTATGGTCGGACCTACCCGACGCGGACAACCCCGCTCATGTGGGAGGC
GACACAGACGCTGCAGAGCTCTCTGGTCGATCAGAGCAGTGACATGCGCCGCTCGCTGGACCTCGCCATG
TCGCTGCACGAGGTGCTGGAGAGAAATCGAGAGATTTACAACAATATCATCGCCGAGCGCAACGAGGCCT
ACCGCCGTCTGCAGGACGCGGACGCGAAACTGCAGCAGGTCGAGCATGTCGTCCGCCGCTACGCCGAGGT
GAAGGATCCGGTGGTGGCCAGCGACGGCTACACGTACGAGCGCACGGAGCTCAGCCGCTACCTTAGCGAC
TGCAAGAAGTCGAACAGCAAGGCCTACTCGCAGCAGACCAAGGAGGAGCTGACAGACGTGATGGTAGACA
ACGTTTCGCTGCGCCGGCTAGCGGAGCTGTTGAAGGGCGTGCACTCGGTGGAGATGCCGCAGCTGTCGAG
CCGCCCGCCGCTAGCGGGCGGTGTTGTCGACGTTAATGGCCCTCGCTCTCACTGGGCCGAGGAGGATCCG
TCAATGGGCGGCGCGGATCACACTGAGATGGGTCCCGGTCCCGTCGGCCTCGCGGGCGGTGCGTGTGGCC
GTGGTGGGAATGGGATGGCAGTGGGTCTCGGTCACGCCGGCCTCCGCTATGATCGGAGCGGAGGTGCCAA
GTCCGGCAGGCCGAGCAACGGCAACGAGGGCAAGGGCGGGCTGCATCCGTGCCTGCGCGTGTATGGTTTC
TGCAACTTCGAGGACGACTGCACGTTTGCCAACTACCCCTACGAGGCATGCCTGAACCATATCAAGGGCA
AGTGTCGCTTCGGCTCCACCTGCAAGGAGCTGCACGTTGACCCGCGCGACCCCGTCTACCAGAGCACACG
CAGCTTTGCCAACCATCACCACCAGGGCGGCAACAGCGCGAACACCAACCACGTCCACAACAACGCCGCT
GCCTGTGCAAACAACGCGAACACCAGCCAGGCAACGGATGTCGGTGCGGAGGCGCCGAGGCAGAGCGGGA
GCAAGATGCCGGACTCTGTAGCGGAACGGGAGACGGAGACTGCGGCGGCCAAGAGAGACGGCAAGTCGAA
GGAGGCCGGGGAGCCCGCTTCTGCTGCAGGTGCAGATGCCGCCAAGGCCAAGGACGAATAAATCGGCGGC
GGAATGGGCTTGTGCATGCGTGCGTGCGTGTGTGTGTGCCGTCTTCATATCTCCACCGACGTCTCCTC
ACTTTTGCACGCCTCGGCCCGCCCTCTCGCGGGCACCCTCTCCCCTGCATTGCACATGCGTGTGTGGCGC
TGCTAGCCTTCGCGCGTAGCGGAAGGGGATGAGGGGGGGGGGGCCTCGCTGACATGACAACGGCAGAGCG
GCAAGAGTGGACGGAGGGCGCCCATTCGCTTTGTTTTTTCCTCTTCCGTTTTCACAGATGACGACACGCC
ACGGGCTCTCTGTGCGCGGGCACATGCCTATGCACGGGCCCCTTCGGCTGGTCACACCGACTCCTGTGGG
GGGCGTGCGTATTCGGTAGAGGATGGTGTGTCCCCTGCCGCCACAGACTCATGGCCGCGTTGCGCCTGTC
GTGGCGCGTGCGGGCAAGCCCCGCAGACGCGCCCCGGTGAAATGGACGGAAATGGAGGATGAAGACAGCG
AGCCCCCGAGACAGCAACGCCGTCCTAAAGCAGGCCCCATGATCCGAAAACGTGAGCGCTGTGTGCACGC
GCTCTCAAGCACAGGTGGCATGGTGTGTCGCATTCACGCAAGGCATCCAGAGGCCGGCAGAGCAAGAGTG
ATGGACGGAGAAGGCGGGAAGGGATCCATGCAAATGGCCCCCCACCCCGCCACACACACACACACACACA
CAATGCATGCGTGCGAAGCATGTGCGGCATGCCCTCCATCTGACCGTGTGGGCTGGCGCGGCGCCGATGC
GGGAAGCACGCCGAGAACACGGCGAGTCGCGCCATCGAGTCCTCCCGGCTCGAATGTGGCGAGCCGCTGC
AGAATCTTGTGGAATGCCGCGGCTTGAGGCGACGGAGGGCGAAGCATGGCATGCGGCCCGGGCACAGGGA
CTCGGAGGCCCTGGCCCGCAGCTGGCCAGCTTCCTGCTTGAGCTCACCAGGCATGCACCTTGACCACCAC
CTGTTATCCTCACAGCGGATGCCACCGTTTCCGGCCTGGGGCAGGACCCTGGGCGCAACCCTCTCGGATC
AAGGCATGCCGTCCTCCCGCGTCTCCGCCTGGCGCTGATCGCGCAGCGCGTATCGGAGAAGGGGACGAGG
AAGGGCGAGAAGAGTCCGTACGACGCACGGCCCGATTCGACGGCAGGGACGGCTACGTCCTGGTCCACCA
GGAGGTAGCTGTGGTGCAGCAGAGGTAGGTGAGCTGCGGTTGTGTCTGCGTGTGTACGTTTATTAACTTG
TGCGCGCGAAGGTGTGGAAAATGTCTTCTCAGCTCTTTCTCATCTCGTTCTCATCTCGTTCTCATCTCTT
TCTCATCTCTTTTGTTTGATTGTGTTTTCCATCGGCTACTCTTGGCGTTTTCGCGTGCGCGTGTGTTTGT
TTGTGCCGGCGTGATGTGGGACAGGAGGGAGAAGACCGCCTGTGTGTTAGCCCCTTTCTCTTGCGTGCTA
GAGTGTTGCGAGATGCGGGCAGATGGTGGGGAGCGACTGTCGGGGACGGCGAAGCTCATGTTCTGGTTGA
CGGTGTGGCGCCTACTCTCTTGCGTTCTCTCTGCACGCGTGCGAATACTTTTTCCTCAGCGTTGTTGATG
Ggcagacggtgcgcaaccgcgtgtggtcgttctctctagcatctgggtctctctaccgccaagtgatgcg
ttcttgcatgtctggtgttatcctcctgtgt

>H145:LmjF.36:2301380-2302271

ccagataggcctgataataatggtgtcgtagtgtgatcttgcggaagttccacttcacctgccgcgacgt
cgttggtgagatgaggtcgagaaggtagtaCAACCTCCAGTTGCGCTTGCGTGGCGGGTGGTCGCAGCAC
ATTCGTCCTCAATGAGCGAAACAGCTGACAGAGATACGACGTGCGGTCACGCAAACTAAACACTCAGACA
ATACGCTCGCCCTCGGTCGACGTGCGCTCACGTCTCTTTCCAACAACGTTGAAGGAGTCCCTCCACAAAG
GGAGGAAGGAGTCAGTCACAGCGACGACAAACAGCGAGACAAAAAAAGGAAGGCTCCTGAAGGGAGAACG
CAGCGAATGCTCTAGAAGAACCAGATTGGTTTCGTTGTTTTTTTTTTCGTTTCGTGTGCGTGTGTGGGTG
AGGGGGTGGGGATGGGGGGGGGGGATGTGGTTACAATGGAACCCAACGGCAACAACGTGCGGCGCACAGT
GAAGTTGGGAACCTCTCCCGCCTCCCAAACGAAAGGAAACAGAAAAAGAAGACACGCACACACAAAGGGC
GGGGGCAGGGGGGGGAGGAAGGGGGGAAGGGGGGAAGGAGGGAAGGCGAAATGCATAGAAAGCGCCAACA
AACGCGGGAGGGAAGAGGGAGGCGGTGAGGCAGGGAAGTCCCCCACCGGTCAGTGAAAGCGAAGGAGGGA
GTTGTGTTGTGGGAAAAGATGATGCGCTTCGCGGCGGAGATGTGAGACGGCAGTGAGCGCCGCAGACGGG
GCAAGGAGAGGGTAGAGGAGAAGACAGGCGGCAACGGCGACAGTACTTTTCTCGAGATCGTCTCTTAGGG
TCTTTTAAAAGCCGAAACGAGCGTCGAGCACAAGAGGCACGTGAGTGTCTGTTCGTGCGCGGTTGTTTGC
GCCTTTTCAGCTTCGCACGGCCGACGTGTCACCAAACAGCAATGACGGTAGCGTGCCGCACGCGGTGCCG
CTGATGGAATTCGGAAAAGGGCGGAAGCGCGGACCCACAGAGATGGTCCGCAAGGCACAAATGGAGGAAA
AGAGCAACGATGATGTCAACGCCAGAGACTCGCGTGTGATCATGTATTCTACTGTACGATTTTCAGGGAG
CAAGATCGGTAGAACGTGCGAGAGAGGGAGAGAAAGGGGGAGCGGCAACACTCACACGCGCATGAACTCG
TCGACGAAGCCCCGCGAACGTGCGTGCTGTTGGGTAAAGTGGTTGAGGCGGGTTCGACGTGTGCACGTGT
GTGAGACTGTCAGAACGTAAGAAGAGAAAAGTGGAGCAGAGAGGAAGAGGAGGTACACACATACACGCAC
GCACATGCAAACAGACAGGTAGAGGGGACGAGATTGGTTTACCGCGGGTGACAGAGGAGAAGCAGGTGAG
CGTCACTGGCAACGTCATAGCGAGAGAGCGAGAGAAGGCGCATCAGCAGTAGGGGAGTCGCACACTGCCG
CGCCATTGCCGTCAGCCTTTTTTTTTTCGTTTTTTCCGCACAACCGCCACAACAAAACTAGACGGTGAAA
CGTCATGCATGGTGAAGTCAAGAATGGAGGTCACAGGACTGGAGGAGAGGGTGTGTGTGCGTGGGGAGGG
GGGGGGGGGGCAGTGGCAGGAAGAGGAGATGGAAAGCCCCCTCTCAGTCTACACCCTTCCCCTCCACGCC
CATCCGACCATGCACCTCTCGTTTCGCGCGGCCCGGCTGCCCAGAGGGGAGCGGTGTATGACACTGTCAC
AGGACGGTCAGCGGTGAGCTTTGTACTGTTCCTGCGTGATGGGATGCTTCCATGTCGTCGACTTCTTGCC
CGACTGTAGAAAGTAGTATTGCATCGTGCGACGCGACAGTCCATACTCCCAGCCGCCGGGAAGAGGCGGA
AACGGGACATCCGCCGCGTGCGTCGCGGCGGCAGTGCTGGCACCGGAAGCAGGCACGGCATTAGCGCCAC
TCGGCGCAGCTGTGCGAGAGTTGTTGGGAGGGCGCGCCGTGGCAGAGCCGGTGGGCTCTGTCGACGAGGC
CGCCGGTGAGCCTGCCAAAGACTCAGTGTTGTTGGCATCGGCTGTCGTCTCTCCCAGGAAGcgaacgggc
agattggaatgagagatggcctgctgccgcgccagccgagcactcgcggcgtccgcaaactgcaccacac
cgtaccggagggcatccacag

>H130:LmjF.27:870624-871831
ctgccgggccactcgccgacccgtcgtggcccccgaaaaagcaccgccacacccgcctgggcgtccttcg
gtgcgagcgactacgcacatcggccccgct**TCAAGCCTCGACCTTCACTGACCACGGGCTCGGCGTCTTG**
CCATACTGGATATCCGTTATCTCGTTCAGGAACCAGCGCGCGTAGCTCGGCTCCGAGAGCGGCACGCTGT
ACTCCTTGCCGCGATACCCAATCATGCTCACGGAAGACACAATGGCAGCCGTGCCGCATCCGAAGCACTC
CATTACGCGCCCCTCCTGCAGCGCCTCCACCAGCTCAGACATGTAGTATGGGCGCTCCGACACCTCGAAC
TCGCCCCACGACCGTGCAAGCGCCAGGATCGAGTCGCGCGTCACGCCGGGCAGGATCGTCCCGTCCAGTG
GCGCGGTGACCAGCTCTGTCTTGCCCTCCTTCGTCTTCCACAGCGTGCAGAAGTTCATGAATCCGGCCTC
CTGCACCTCATCATTGGAGCCGAGCCACAACACCTGGTTGTAGCCGAGAGCCTGCGCCTCCTCCTGAACC
AGTAGCGGCCCAGCGTAGTTGGCACCCAGCTTGCTGCCGCCAGTGCCACCGGGCCACGCGCGCTTGCGCT
TATCCTCCGCCAGCAGCGTCACGGGCTTGATGGCCATCGCACCCGACTGACCCTCCGGCGGCAGGTAGTA
CGGCCCCACGGGCGAAGCAATCACGAAGAGACGCACCGACCTCGAGGGAGTGGCGCCAAGGCTATCTGCG
GTGCCGATAGCAGCCGGACGCAGGTAAAGCGAGTAGCCGTGCTCCTTAGGCACGTAATCCTTCTCCGTCC
TCACGAACTCCGTGATCACCTTCAGCATCTCCGCCTCGTCAAAGTCCGGGAAGCACAGAGAGCGCATGCT
GTGGCGCAGGCGCGCAACGTTCCGGTCAGGGCGGAACAGGCGCAGGCTCTGCTTCTCCAGCTTCTCACCC
CTCGCGGCCCTCGTGATATCAGCAATGTCCGCGTACACCTTCATTCCCTCAAAGCACTGTGTCGCGTAGT
GCAGGCACGACGTCTGCGGCGGCAGCGAAAAGTTGGCGAAAGGTACGATCGCCGGCGCACCCCACCTGCC
GTCGCTGAAGTCGATTATCACCATGTGCGGCGAAAAGAGGGTGCCAAAGGCGACGCCCTTCATCGGCGGC
AGCGGCGGCGGATCGGTCACCAGTGTTTTAGTAAGCGCCGCAGCCGTGAAGGAGACGACGGGGGCCCTGC
TGCCACGCGCAGCGCTGGCCTGGTGCCAGAGTCGGCTCAGCAACATGGCAAGAAGTCTAGGGGGGGGCGC
TGCCAGAGCAGAGGGGAAAAGACAGAGATGTGGACAGTCGATGCTGGGAGCGGGATGACTCAATCAACCA
GAGCGGCGAGTGATGATGATATCGTTGGCCTGTGAAGCTGGTGCAACGACGACCAGTGCATGTGTGTGTG
GTGTGCTTGGAGGATGGATGTACGATGATCTGTCCAACGGGAGACAAGGAGCGGTGCAGAAGCATAGCGA
GGTAGGGAGGTCGGTAGCAGCAATGATGGAGAAGGAGAGGGACATGCAGCGAAAGAGAGCACGGTCGAGA
ATACGCATACGCACATACACCCACACAGGATACCAGGCAGTCGCGAGTGCGAGCATGCCGCACCCTGCAC
GCGCTGCTAGACCGAAGCCGGCGACGAGGTTCCGTAGGCGAGCCAAATCGCACCGCTTTCAACAAGCAGG
GGGTGCACCGCACTGAAATGCACGTCTTCGTGGGCAAAGATGGCGCGCGTGTGTCCGTGTAAGGCAAGCG

GGGCGGGACATGGGAAGGCGGTGGCCGTTCGCTCCTTGCCGCGCCTCTCCTGTGCTCCTTGTCTTTGCGG
TTGGTGCTTCTGCCTTGCGTGTAGCCCGCGCCTCATCGCCCTCCCTCTCTCCGCGGGCAGGCGCGACTTC
GCTTGCCCTTTGTGCGCTGATGCCTGATGGCGTCCGTGAATGCAGCACTTCCATTCCGATTGCTGTCGCG
CGAGAGACTCAGAAGACAGGCAGCAAAAGGGAGAGAGAAAGAGAGCGCCTAACAACCGAAGCATGTGTGT
GTGTGGTGTGTGTGTGTGTGTATGTGTGAAAGAGAAATGTAGATGATCGTCTTTCGGCATGAGAGCTCCG
GTGAGCGAAGAGGCGCACACACATTCGCACGCACGTAAAGAGTCGCCTACGCAGAATGGCTGCAGCAGCC
TCCTCCCTCCCCCCCCCGTTTCACACACACACACACACACACACACCTGCGCACACACACACACCGCT
ACTGCGTGCTGCTCTGGACCCTCGCACAGCTGAGCTCCAGGGGCGCCAACAAGCACCCGTGTGGGACTCC
CTGCTGTCCCGTTCTTCTTCGCACACAGAGAGAGACGTTCCTTACACAAAGGTAAGCAAGGGGGTCCACT
TGAGGTACGAATAAAAGAAAAGAAAGCAAAGTTCGGTTAGCGGAACGAAAAGAGAAGTCGCGATCTCGCG
CGAGAGAGAAACCTGAAGTTACAGCATCGCTTCATCGGGGCGGTGTTCATCCTCGGTGCCGCGATCGCCG
ACAAGAGCGAGAGGAAAGACAAGAATGACAAAACAAAAGGGTTGTAGGTGAGTGAGGGATTAGCGTGTGT
GAAAGGGCGGCAAAGGGAGGCCAAGTCGGTTGGGGTCGGGGGAAAGAAGAGAGTGAAACGATGGAGTGAA
GTAGCCGGCGCGCTTCCCACCTCGCCAGAACACCACCCACCGCCCTTCGCCTCGCTGAATCAGCACCACA
ATGCCCGCCGACATCACCATCAGCAGCGAGAGCAGCCGTATGCTCGTCGCGAGTTCGACGACGGAACAGT
CGATGATGGCGGCGGCCTGCCCCTCCACTCCACGACCTGCCACTTCCGAGTGGGCGAAGAGGCGGCGAGC
CGGAGTAGAGATAGATATAGCAATATACCTATACAGAGGGAAAACGATACATAGAAAAGATCGTGCGAGA
GGTCAATGAAAGTAGGTCACAACACAAAAAATGAACGTCTTTTGGAGTGAGAGAAAAGCTCGGTGGTGGA
GGCAGACAGCGATGCCCGGAGGAGGAGGAGGAGGAGGAGGAGGAGGAGGAGGAGGAGGAAGAGGAGG
AGGGAAAAGGAGGGAGGAGAACGCCTATGCGCCAGCACACACCGCGGGTCAAGCAAAAGGAGATGAACAC
ACGTGCACGTGAGTCCACCGCGAGTACACATTAGCCACTCGCACACACACACACACGCGCACACAACAAC
AATTTTTGGAACATGAAAAAGAACGGAGCACGCGTGTGTGGTATGTGTGCGTTAAACAAGAACGAAAGAG
AGGGGGAGGGCGCGGTCAGGCAGGGGTTCCTCGGCGTTCATACACGTAACAACTTTGACGAGCACCGACA
AATTTCCACACCCCTGCTCACCAGCGGGGTACAAACAACAACGATACCAAGAAAAAAAAACGTGCACGCA
CACACACCAGTAATAGCAATCGTGAAAGACGGTTAGCGGGGAGGGGGAAAAGACGATGATTGTCGTGGTC
ATGGTCATGGAGGGGGGAGATCGCACACGATGGACGCAGCACACCCACGTAAAACACGGAACCCAGGCGC
GCGCATCTCATCTATTAGCTTCTCATCGTCTTAAGGATGTCACACACGCACGTGTGCGACTCTCTGTATA
ACTAGCAAGGCGCCCTGGCACTTGCCTGCCCCTCTTGCCCACATCATCGCCACACCCGCCTGGGCGTCCT
TCGGTGCGAGCGACTACGCACATCGGCCCCGCT**TCAAGCCTCGACCTTCACTGACCACGGGCTCGGCGTC**
TTGCCATACTGGATATCCGTTATCTCGTTCAGGAACCGGCGCGCGTAGCTCGGCTCCGAGAGCGGCACGC
TGTACTCCTTGCCGCGATACCCAATCATGCTCACGGAAGACACAATGGCAGCCGTGCCGCATCCGAAGCA
CTCCATTACGCGCCCCTCCTGCAGCGCCTCCACCAGCTCAGACATGTAGTACGGGCGCTCCGACACCTCG
AACTCGCCCCACGACCGTGCAAGCGCCAGGATCGAGTCGCGCGTCACGCCGGGCAGGATCGTCCCGTCCA
GTGGCGCGGTGACCAGCTCTGTCTTGCCCTCCTTCGTCTTCCACAGCGTGCAGAAGTTCATGAATCCGGC
CTCCTGCACCTCATCATTGGAGCCGAGCCACAACACCTGGTTGTAGCCGAGAGCCTGCGCCTCCTCCTGA
ACCAGTAGCGGCCCAGCGTAGTTGGCACCCAGCTTGCTGCCGCCAGTGCCACCGGGCCACGCGCGCTTGC
GCTTATCCTCCGCCAGCAGCGTCACGGGCTTGATGGCCATCGCACCCGACTGACCCTCCGGCGGCAGGTA
GTACGGCCCCACGGGCGAAGCAATCACGAAGAGACGCACCGACCCCGAGGGAGTGGCGCCAAGGCTATCT
GCGGTGCCGATAGCAGCCGGACGCAGGTAAAGCGAGTAGCCGTGCTCCTTAGGCACGTAGTCCTTCTCCG
TCCTCACGAACTCCGTGATCACCTTCAGCATCTCCGCCTCGTCAAAGTCCGGGAAGCACAGAGAGCGCAT
GCTGTGGCGCAGGCGCGCAACGTTCCGGTCAGGGCGGAACAGGCGCAGGCTCTGCTTCTCCAGCTTCTCA
CCCCTCGCGGCCCTCGTGATGTCAGCAATGTCCGCGTACACCTTCATTCCCTCAAAGCACTGTGTCGCGT
AGTGCAGGCACGACGTCTGCGGCGGCAGCGAAAAGTTGGCGAAAGGTACGATCGCCGGCGCACCCCACCT
GCCGTCGCTGAAGTCGATTATCACCATGTGCGGCGAAAAGAGGGTGCCAAAGGCGACGCCCTTCATCGGC
GGCAGCGGCGGCGGATCGGTCACCAGTGTTTTAGTAAGCGCCGCAGCCGTGAAGGAGACGACGGGGGCCC
TGCTGCCACGCGCAGCGCTGGCCTGGTGCCAGAGTCGGCTCAGCAACATggcaagaagtctagggggggc
gctgccagagcagaggggaaaagacagagatgtggacagtcgatgctgggagcacagtgctgatgtttat
gggcgagtg
